# A multidimensional narrative review of the association between air pollution and late-life depression risk

**DOI:** 10.3389/fpubh.2026.1813992

**Published:** 2026-04-22

**Authors:** Xiaofen Zhang, Xinrui Yang, Yao Zhao, Kaida Chen

**Affiliations:** 1 School of Basic Medical Sciences, Xiamen Medical College, Xiamen, China; 2 College of Landscape Architecture and Art, Fujian Agriculture and Forestry University, Fuzhou, China

**Keywords:** air pollution, environmental exposure, late-life depression, public health, urban-rural environment

## Abstract

Synthesizing evidence from 80 empirical studies identified through a systematic search of Web of Science, this review highlights the association between air pollution exposure and late-life depression. Results indicate that exposure duration is a critical modifying factor: acute, short-term exposure correlates with symptom exacerbation, whereas long-term, cumulative exposure is linked to increased incidence. Pronounced geographical heterogeneity emerges, with larger effect estimates consistently reported in highly polluted regions. Critically, significant associations persist even in low-pollution settings like Sweden, challenging the notion of a safe exposure threshold. Furthermore, neurotoxic effects vary markedly across pollutants, with PM_2.5_ (and its specific components), NO_2_, O_3_, and indoor solid fuel combustion demonstrating particularly pronounced associations. Potential synergistic effects between indoor and outdoor sources are also suggested. In conclusion, air pollution constitutes a significant and modifiable environmental determinant of late-life depression. Future efforts should integrate multidimensional exposure assessments into urban planning and public health policy, implementing targeted interventions for vulnerable populations to simultaneously improve environmental quality and mental health in later life.

## 1 Introduction

### 1.1 Background

The accelerating global shift toward an aging population underscores late-life depression as a pivotal challenge in both public health and clinical practice. Beyond its core manifestations of subjective distress, social withdrawal, and reduced quality of life, depression in older adults (≥60 years) exhibits robust associations with cognitive decline (1), cardiopulmonary diseases (2), and other chronic conditions. These comorbidities collectively amplify the disease burden and care demands within this growing demographic (3). Neurobiologically, depression and neurodegenerative disorders such as Alzheimer's disease share overlapping pathophysiological mechanisms, a convergence that may indicate early neurological dysfunction or accelerate cognitive decline through pathways involving chronic inflammatory stress (1, 4). Given these shared pathways, identifying and modifying external, modifiable risk factors represents a vital strategy for preserving brain health in aging populations.

Among modifiable external risk factors, air pollution has gained prominence in epidemiological research due to its dual role as a pervasive environmental exposure and a potential neurotoxicant affecting central nervous system health. Substantial evidence now links both acute (short-term) exposure to high-pollution episodes and chronic (long-term) ambient exposure to an elevated risk of depression in older adults. Short-term exposure to pollutants including, fine particulate matter (PM_2.5_), nitrogen dioxide (NO_2_), and ozone (O_3_), is consistently associated with acute exacerbations of depressive symptoms and increased emergency department visits (5, 6). In parallel, sustained exposure to these pollutants demonstrates a strong relationship with the incidence of late-life depression and clinically diagnosed late-onset depression (7–9).

To contextualize these findings, it is essential to consider established air quality guidelines. According to the World Health Organization's (WHO) 2021 Global Air Quality Guidelines, the recommended annual mean concentration limits for PM_2.5_ and NO_2_ are 5 μg/m^3^ and 10 μg/m^3^, respectively, with corresponding 24-h limits of 15 μg/m^3^ and 25 μg/m^3^ (10). These values represent the exposure levels above which clear evidence of adverse health effects exists, and are substantially lower than previous guidelines (e.g., PM_2.5_ annual limit was 10 μg/m^3^ in the 2005 WHO guidelines). Importantly, as emphasized by the WHO and expert bodies such as the UK Committee on the Medical Effects of Air Pollutants, these guideline values should not be regarded as thresholds below which no health effects occur; rather, current evidence supports a linear, no-threshold relationship, meaning that even concentrations below these levels may still pose risks to human health (11). This understanding is particularly relevant when interpreting findings from low-pollution regions.

Notably, these associations display significant spatial heterogeneity. Effect estimates tend to be larger in highly polluted, developing regions (12). Conversely, cohort studies from areas with relatively clean air, including Sweden and Ireland, corroborate that chronic low-level PM_2.5_ exposure still correlates with increased depressive symptom risk among older adults (13, 14). This pattern of association across diverse exposure gradients suggests a heightened neurological susceptibility to pollutants and directly challenges the traditional concept of a safe exposure threshold. Furthermore, the indoor environment represents a critical, yet often overlooked, exposure domain. Sustained utilization of solid fuels for household cooking or heating—a principal origin of indoor air pollution—serves as an autonomous risk determinant for depression among older adults (15–17), highlighting the potential for interactive effects from coexisting indoor and outdoor pollution sources.

From a neurobiological perspective, air pollution contributes to depression pathophysiology via multiple, interacting direct and indirect pathways. Direct mechanisms involve the inhalation of ultrafine particles and toxic gases, which induce systemic oxidative stress and chronic low-grade inflammation. Such inflammatory and oxidative mediators may enter the central nervous system either via a compromised blood-brain barrier or through neural routes like the olfactory nerve. Once inside, they activate microglia, promote neuroinflammation, and disrupt key neurotransmitter systems, including dopamine and serotonin signaling (18, 19).

The established overlap between depression and neurodegenerative disorders underscores the importance of shared pathological pathways, particularly those involving chronic neuroinflammation. Air pollutants, most notably fine particulate matter (PM_2.5_), serve as powerful exogenous stimuli that can activate and sustain this inflammatory cascade. The mechanistic sequence begins with pollutant inhalation, inducing systemic oxidative stress and pro-inflammatory cytokine release (e.g., TNF-α, IL-6). These peripheral mediators can then translocate to the central nervous system, where they promote microglial activation and persistent neuroinflammation. This environment disrupts neurotransmitter systems, impairs synaptic plasticity, and suppresses hippocampal neurogenesis—core processes implicated in depression pathophysiology and often observed in early neurodegenerative changes. Consequently, air pollution emerges not merely as a respiratory hazard, but as a neurotoxic stressor capable of exacerbating or triggering the inflammatory pathways that underlie both late-life depression and cognitive decline, thereby bridging environmental exposure with deteriorating mental health in aging populations.

Concurrently, exposure to air pollutants can dysregulate the hypothalamic-pituitary-adrenal (HPA) axis, a core pathophysiological component of depression (9). Beyond these direct biological insults, indirect pathways further elevate depression risk. Air pollution can induce or exacerbate cardiometabolic and respiratory diseases, thereby compromising general physical health—an established vulnerability factor for depression (20). Additionally, it can directly impair sleep architecture and quality, disrupting essential physiological and neurobiological recovery processes (21, 22). Pollution-related physical symptoms or avoidance behaviors often lead to reduced physical activity and increased social isolation, diminishing psychological resilience and heightening depression susceptibility (23, 24).

Guided by this mechanistic understanding, the research frontier has progressively shifted toward identifying practicable intervention strategies. A growing body of evidence indicates that individual- and household-level interventions—such as improving indoor ventilation (25, 26) and transitioning from solid to clean cooking fuels (24, 27)—can directly alleviate depressive symptoms. At the community and urban planning level, increasing access to green space not only provides independent mental health benefits but also moderates the detrimental impacts of air pollution on depression (28, 29). At the broadest policy level, nationwide initiatives like China's “Healthy City” and “New Energy Demonstration City” programs serve as naturally occurring quasi-experiments, demonstrating that comprehensive environmental management can effectively reduce population-level depression (30–32). These multi-level findings provide a novel perspective on the prevention of neuropsychiatric disorders, outlining a practical blueprint for intervention. They underscore that integrated environmental and policy interventions can achieve dual objectives: reducing risk exposure at its source while simultaneously promoting mental health in older adults and supporting environmental sustainability.

### 1.2 Significance

Although existing studies have confirmed the association between air pollution and late-life depression, the evidence remains largely fragmented—often focusing on isolated exposure scenarios or singular mechanisms—and lacks a comprehensive synthesis of multi-dimensional factors. Current epidemiological data robustly and consistently associate sustained exposure to pollutants including PM_2.5_ (7, 33) and NO_2_ (8, 34) with heightened late-life depression risk, while also verifying that acute exposure can precipitate symptomatic worsening (5, 6). Research into underlying pathophysiological mechanisms is advancing rapidly, with prevailing models positing that air pollutants, through systemic inflammation and oxidative stress, impair blood-brain barrier function, activate microglia, and contribute to neuronal dysfunction (9, 18, 35). These core biological pathways interact with modifiable behavioral and social factors—including sleep disturbances (15), altered physical activity pattern (34, 36), and diminished social support networks (13)—forming integrated pathways to depression. Moreover, the field is increasingly embracing a translational perspective, with accumulating evidence assessing how environmental modifications—such as increased exposure to green space (28, 29) and the adoption of clean energy policies (24, 30)—influence depression risk. In parallel, a critical focus on environmental justice has uncovered significant disparities: older adults in vulnerable subgroups (e.g., rural residents, individuals with lower socioeconomic status) bear a disproportionate burden of pollution-related depression risk, likely stemming from both greater cumulative exposure and potentially increased biological susceptibility due to pre-existing vulnerabilities (12, 37, 38).

Recent systematic reviews have explored the association between air pollution and depression—for instance, the meta-analysis by Borroni and colleagues focused on overall PM_2.5_ exposure (39), and the updated review by Bereziartua and colleagues extended to anxiety outcomes (40). However, these studies have largely been confined to a single dimension and have not systematically integrated the interactions among exposure duration, geographical context, and pollutant characteristics. However, these studies have largely been confined to a single dimension and have not systematically integrated the interactions among exposure duration, geographical context, and pollutant characteristics. Consequently, this fragmented evidence base impedes a holistic understanding of the risk continuum from environmental exposure to depressive symptoms and hampers the formulation of precisely targeted public health strategies.

To address these critical gaps, this review provides a comprehensive retrospective synthesis and critical appraisal of the existing evidence through an integrated neuroepidemiological and geriatric psychiatry lens. It aims not only to consolidate established associations but also to systematically identify consistencies, discrepancies, and reconcile potential contradictions in the evidence across key dimensions. Specifically, the review delineates the distinct neurobiological mechanisms that may underlie depression associated with short-term acute vs. long-term chronic exposure, contrasting transient stress-axis activation, and neurotransmitter imbalance with mechanisms involving sustained neuroinflammation and cumulative impairment of neural plasticity. Furthermore, it examines the spatial heterogeneity in exposure-response relationships by comparing findings from high- and low-pollution regions, thereby contributing to critical debates on establishing neurotoxicity thresholds and developing protective standards (13, 14). Finally, the review systematically evaluates the differential neurotoxic potential of various pollutants based on their physicochemical properties—including specific PM_2.5_ constituents (35, 41), gaseous pollutants, and indoor emissions from solid fuel combustion (15, 16)—with a particular emphasis on elucidating the combined effects of concurrent exposure to multiple pollution sources from both indoor and outdoor environments.

By synthesizing evidence across exposure duration, geographical context, and pollutant characteristics, this review aims to clarify the role of air pollution—an important environmental neurotoxicant—in the development and progression of late-life depression. This synthesis will help bridge the current fragmented understanding of the underlying pathophysiological links. It also suggests promising avenues for future research, including the development of more precise exposure biomarkers, the application of neuroimaging to identify vulnerable brain regions and intermediate phenotypes, and the exploration of targeted intervention strategies addressing specific mechanisms such as neuroinflammation.

## 2 Literature search and framework

### 2.1 Data sources

A systematic search for relevant literature published between 2020 and 2025 was conducted on December 15, 2025, using the Web of Science database platform (including the Science Citation Index Expanded, Social Sciences Citation Index, and Arts & Humanities Citation Index). The search strategy combined keywords across three key domains: (1) environmental exposures (“air pollution”, “PM_2.5_”, “PM_10_”, “nitrogen dioxide”, “ozone”, “green space”); (2) mental health outcomes (“depression”, “depressive symptoms”, “CES-D”, “PHQ-9”, “GDS”); and (3) target population (“older adults”). The initial search identified 147 potentially relevant records, aiming to systematically retrieve contemporary epidemiological studies investigating the link between air pollution and depression in older adults, thereby establishing an evidence base for subsequent multi-dimensional analysis.

For transparency and reproducibility, the complete search strategy executed in the Web of Science database is presented below:

**Search String:**

TS = (“air quality” OR “particulate matter” OR “PM_2.5_” OR “PM_10_” OR “nitrogen dioxide”)

AND

TS = (“depression” OR “depressive symptoms”)

AND

TS = (“older adult”)

Refined by: [Publication Years: 2020–2025] AND [Document Types: Article]

Databases: Web of Science Core Collection (SCI-EXPANDED, SSCI, A&HCI)

Date Searched: December 15, 2025

Records Identified: 147 → 130 (after limiting to Article document type)

It should be noted that this review searched only the Web of Science database and did not include other major biomedical databases such as PubMed or Embase, which may have resulted in the omission of relevant publications. Moreover, the search terms focused primarily on “depression” and common assessment tools, without fully covering other related mental health outcomes (e.g., anxiety, psychological distress), potentially limiting the scope of identified literature. These limitations should be considered when interpreting the findings of this review, and future research employing more comprehensive search strategies is warranted to validate and extend our conclusions.

### 2.2 Screening process

**Figure 1:**
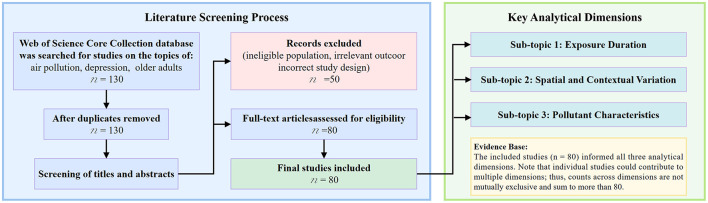
Literature screening.

A standardized, multi-stage screening protocol was followed. First, the search results were limited to the “Article” document type, yielding 130 records, which were imported into EndNote 20 for deduplication; no duplicates were identified. Following this, the 130 records underwent sequential evaluation based on titles and abstracts against the predefined inclusion and exclusion criteria. The initial title/abstract screening was conducted by one author, with a random sample independently reviewed by a second author to ensure consistency. Studies deemed potentially eligible then proceeded to full-text assessment. The full-text screening confirmed each study's relevance to the review's multidimensional framework (exposure duration, geographical context, and pollutant type) and its methodological appropriateness for narrative integration. Exclusion criteria encompassed studies with ineligible populations, non-relevant outcomes, ill-defined exposures, or non-primary research literature (e.g., reviews, systematic reviews, meta-analyses). Any discrepancies or uncertainties identified during screening were resolved through discussion between the authors until consensus was reached. Through this structured process, 80 studies were selected to form the core evidence base for this review (Figure 1).

It should be noted that although a systematic literature search and screening process was employed, substantial heterogeneity across the included studies—in terms of exposure definitions, outcome measurements, population characteristics, and study designs—precluded a quantitative meta-analysis, as such pooling might yield biased effect estimates. Therefore, a narrative integration approach was adopted to qualitatively integrate and comparatively analyze the evidence across multiple dimensions, rather than providing a single pooled effect estimate. The final set of literature encompasses a wide variety of pollutant types, geographical settings, and methodological designs, providing a solid epidemiological foundation for the subsequent multi-dimensional analysis and narrative integration that is the focus of this work (42–44).

### 2.3 Basic characteristics and evolution trend

The included studies exhibited distinct temporal and geographical patterns. Temporally, a steady increase in publication volume is evident over the last 5 years, with a particularly pronounced surge between 2024 and 2025, indicating rapid knowledge expansion in this research field (30, 42, 45, 46). Geographically, the evidence base encompasses regions with widely varying air pollution levels and socioeconomic profiles. Studies from China constitute a substantial proportion, reflecting the co-occurring challenges of rapid population aging (growing proportion of adults ≥60 years) and significant environmental pollution in this context (5, 16, 30, 33). Concurrently, important contributions come from high-income regions in North America and Europe (e.g., the United States, the United Kingdom, Sweden (7, 13, 47) and from other parts of Asia, such as South Korea and Taiwan, China (18, 28, 34). Collectively, these studies provide essential cross-regional comparisons that aid in discerning both the generalizability and context-specific variations of the air pollution-depression association.

The methodological landscape of this research field has evolved significantly, marked by increasing diversification of study designs. Foundational evidence emerged from early cross-sectional studies that established initial associations between pollutant exposure and depressive symptoms (5, 15, 25). This was followed by prospective cohort studies, which utilized long-term follow-up data to provide more robust evidence for temporal, and potentially causal, relationships (13, 35, 43). In parallel, case-crossover designs have been employed to pinpoint the acute effects linking short-term pollution exposure to depression-related outcomes (41, 47, 48). Most notably, the recent proliferation of quasi-experimental studies (24, 27, 31) represents a pivotal expansion of the research paradigm—shifting the focus from merely identifying environmental risks toward actively evaluating the real-world effectiveness of existing environmental policies and interventions.

### 2.4 Framework

**Figure 2:**
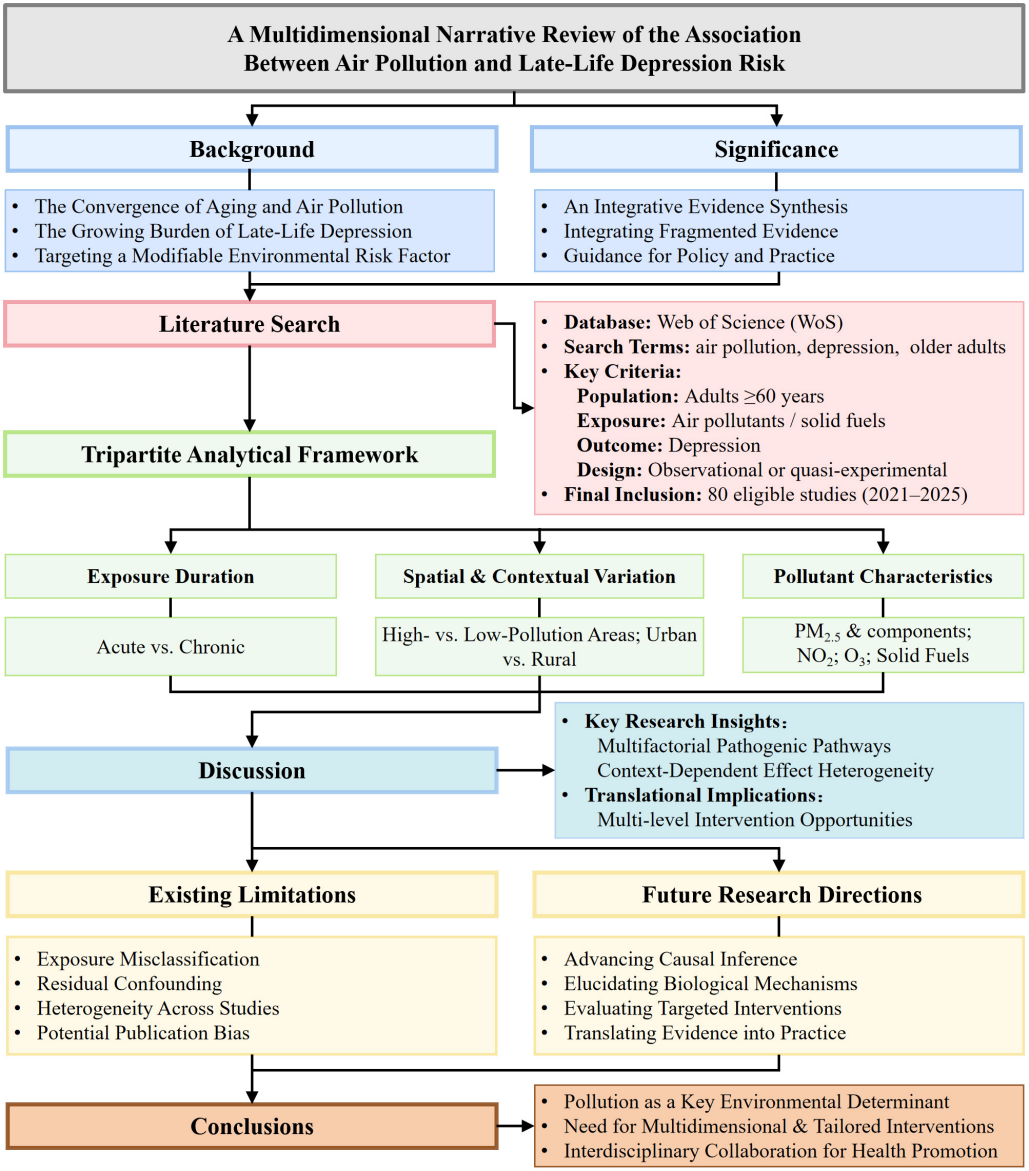
Conceptual framework.

The analytical framework of this review is illustrated in Figure 2. As a narrative review, this paper first establishes the research background and core questions, followed by a detailed description of the literature search and screening process. The core of the review involves the organization, comparison, and integration of epidemiological evidence structured along three principal dimensions: exposure temporality (acute/short-term vs. chronic/long-term), geographical context (including regions with varying pollution levels and urban-rural distinctions), and pollutant characteristics (e.g., PM_2.5_, NO_2_, and household fuels). On the basis of this synthesized evidence, the review then discusses the potential underlying neurobiological and psychosocial mechanisms, as well as the heterogeneity observed across studies. Subsequently, it addresses current methodological limitations and knowledge gaps, suggests directions for future research, and presents integrated conclusions. It is crucial to clarify that this work represents a qualitative synthesis and critical appraisal of observational evidence, not a quantitative meta-analysis. This tripartite analytical framework—focusing on time, space, and pollutant type—was selected to systematically delineate the patterns of association, key effect modifiers, and relevant biological and psychosocial pathways, thereby offering a coherent and comprehensive synthesis of this complex relationship, as the existing evidence base is primarily concerned with elucidating the epidemiological association between air pollution and late-life depression.

### 2.5 Key analytical dimensions

An integrative analysis of the evidence, structured by the established conceptual framework, reveals three central and interconnected themes that characterize contemporary research on the air pollution–depression association in older adults. These themes collectively form the evidentiary foundation and define the primary analytical axes of the present review.

Regarding the temporal dimension of exposure, evidence points to distinct underlying mechanisms for the effects of short-term vs. long-term exposure. Research on short-term exposure (typically defined as lasting from days to 1 year) focuses on the immediate link between transient pollution spikes and the onset or worsening of depressive symptoms. For example, studies have examined how variations in PM_2.5_ moving averages over specific exposure windows correlate with depressive symptom scores (5), or have assessed the impact of short-term O_3_ exposure (6) and specific PM_2.5_ components (41) on the risk of outpatient visits for depression. In contrast, studies of long-term exposure—generally covering periods exceeding 1 year—investigate the association between sustained pollutant concentrations and depression risk, drawing on longitudinal data from cohorts in diverse regions such as China (16, 33, 35), the United States (8), and Sweden (13). A limited but methodologically valuable set of studies directly compares both the short-term and long-term effects of the same pollutants, offering an integrated perspective (49).

Concerning the spatial and contextual dimension, heterogeneity in findings is strongly influenced by variations across regions in pollution levels, socioeconomic conditions, and demographic structure. This heterogeneity is illustrated, for instance, by comparative studies conducted in areas with markedly different pollution concentrations (14, 50). Moreover, urban-rural disparities represent a key modifier of risk. Research indicates that differences in primary exposure sources and living environments contribute to distinct risk profiles for depression among older adults in rural vs. urban settings (37, 38, 51). Furthermore, at the community level, specific environmental attributes and the outcomes of policy interventions have become vital avenues for investigating the potential of environmental modifications to mitigate risk. These include the quality of the built environment (52, 53), the accessibility and characteristics of green spaces (28, 42, 54), and the demonstrated effects of targeted policy programs like “Healthy Cities” (31) and “New Energy Demonstration Cities” (24).

Investigations into pollutant characteristics have evolved from examining aggregate measures toward disentangling specific constituents and sources. PM_2.5_, the most widely studied pollutant, demonstrates robust associations with depression across multiple epidemiological designs (7, 8, 13, 35). A shift toward compositional analysis is evident, with research seeking to apportion risk to specific PM_2.5_ components—including black carbon, sulfate, and organic matter—and to quantify their relative contributions (35, 41, 55). Concurrently, the independent roles of gaseous pollutants such as NO_2_ (8, 19, 34) and O3 (6, 8, 9) have been rigorously assessed. Importantly, indoor emissions from solid fuel combustion constitute a distinct exposure domain, which has spurred focused research on its links to depression (16, 23, 30) and the elucidation of underlying mechanistic pathways (15, 22).

In summary, the current body of evidence has been accumulated and deepened precisely through the investigation of three principal dimensions: the duration of exposure, the geographical and socioeconomic context, and the specific attributes of pollutants. These three core dimensions form the analytical framework that guides the detailed synthesis and comparative analysis of the epidemiological evidence presented in the following section.

## 3 Association between environmental exposure and depression risk

### 3.1 Exposure duration

The duration of exposure is a key determinant of the association between air pollution and depression, leading to distinct risk patterns for short-term (acute) and long-term (chronic) exposure. Evidence reveals a stable and cumulative positive association between long-term exposure (typically defined as ≥1 year) and increased depression risk, consistently confirmed by large prospective cohort studies worldwide. For instance, a U.S. Medicare study found that chronic exposure to PM_2.5_ (HR = 1.009, 95% CI: 1.000–1.018), NO_2_ (HR = 1.006, 95% CI: 1.003–1.009), and O_3_ (HR = 1.021, 95% CI: 1.016–1.026) was associated with higher risk of diagnosed late-life depression among older adults (≥65 years) (8). Research in China has similarly linked prolonged exposure to various pollutants (including PM_2.5_, PM_10_, SO_2_, and CO) with elevated depression incidence. For instance, each 10 μg/m^3^ increase in PM_2.5_ was associated with a 42% higher risk (HR = 1.42, 95% CI: 1.35–1.50) (56). Remarkably, even in regions with relatively low pollution levels, such as Sweden (13) and Ireland (14), studies have detected significantly increased risk among older adults associated with chronic PM_2.5_ exposure, despite annual mean concentrations being well below international air quality guidelines. In the Swedish cohort, each 1 μg/m^3^ increase in PM_2.5_ was associated with a 55% higher risk (HR = 1.55, 95% CI: 1.22–1.93). This indicates a particular sensitivity of the nervous system to pollution, suggesting that cumulative exposure at low levels still poses a mental health risk and challenging the traditional concept of a safe exposure threshold (50).

In contrast to the chronic effects of long-term exposure, short-term or acute exposure (spanning hours to weeks) is associated with the acute exacerbation of depressive symptoms and increased utilization of related healthcare services (e.g., outpatient or emergency department visits), often exhibiting a lagged effect. The case-crossover study design has been instrumental for providing robust evidence of this short-term association. For example, multiple studies conducted in Chinese cities have found that short-term exposure to PM_2.5_ and certain of its chemical components can increase the risk of outpatient visits for depression. Specifically, PM_2.5_ exposure was significantly associated with outpatient visits (OR = 1.607, 95% CI: 1.321–1.956), with similar effects observed for its organic matter (OR = 1.417, 95% CI: 1.245–1.612) and sulfate components (OR = 1.418, 95% CI: 1.247–1.613). The effect often peaks after a specific lag period (e.g., 21 days) (41, 43). Additional evidence from California, USA, confirms that short-term O_3_ exposure is associated with an elevated risk of emergency department visits for depression, with a 1.87% increase under a 7-day cumulative exposure window (95% CI: 0.62%−3.15%) (57).

Moreover, the strength of the association exhibits dynamic changes depending on the length of the exposure time window. This is exemplified by a nationwide cross-sectional study in China, which reported that the association between PM_2.5_ exposure and depressive symptoms varied with exposure window duration, showing the strongest association when using a 180-day moving average (5). In another multi-province study in China, medium-term O_3_ exposure, quantified as a 3-month moving average, was positively associated with the risk of both depression and anxiety (OR = 1.17, 95% CI: 1.08–1.27). Furthermore, this study found that higher ambient temperatures enhanced the specific association between O_3_ exposure and anxiety risk (6).

These findings point to duration-dependent pathogenic pathways. Short-term exposure may mediate immediate effects via acute neuroinflammation, oxidative stress, and transient stress-axis dysregulation. Long-term exposure likely promotes sustained pathophysiological states—persistent neuroinflammation and cumulative neural plasticity impairment—or influences health behaviors chronically. Translating this temporal distinction into clinical and public health practice, the identification of critical exposure windows is essential for developing accurate early-warning and intervention frameworks. Operationally, this entails that surveillance of short-term pollution fluctuations can facilitate timely alerts for acute symptom onset, while sustained reduction of long-term exposure constitutes a cornerstone strategy for alleviating the population-level burden of depression. Overall, evidence regarding exposure duration is consistent: both short-term and long-term exposure are positively associated with depression risk. Short-term studies predominantly employ case-crossover designs, offering advantages in controlling time-invariant individual confounders, while long-term studies are mainly derived from prospective cohorts with stronger causal inference capabilities. The complementarity of these designs strengthens the reliability of evidence in this dimension.

### 3.2 Spatial and contextual variation

The association between air pollution and late-life depression exhibits marked spatial heterogeneity. This heterogeneity stems from large-scale differences in regional pollution levels and, more profoundly, from fundamental contrasts between urban and rural living environments and their predominant pollution sources.

In highly polluted developing regions, such as China, multiple nationwide cohort investigations have consistently demonstrated a strong association between long-term PM_2.5_ exposure and an increased risk of late-life depression, with hazard ratios commonly ranging from 1.2 to 1.5. For instance, one study reported that each 10 μg/m^3^ increase in PM_2.5_ was associated with a 42% higher risk of depression (HR = 1.42, 95% CI: 1.35–1.50), while another found an even stronger association for black carbon components (HR = 1.54, 95% CI: 1.44–1.64) (12, 35, 56).Concurrently, the widespread household solid fuel use in these areas represents a significant independent exposure source. Prolonged solid fuel use for cooking (HR = 1.26, 95% CI: 1.13–1.40) or heating (HR = 1.27, 95% CI: 1.14–1.42) substantially elevates depression risk in older adults (16, 17), often through deteriorated sleep quality and physical health (15, 23). A systematic review further concludes that pollutants emitted from burning solid fuels are key environmental factors contributing to depression, anxiety, and related mental health issues among populations from low- and middle-income countries (58).

Conversely, prospective research conducted in European and North American settings with comparatively low ambient pollution has consistently established positive associations between long-term air pollutant exposure and depression risk, even at exposure levels well below international air quality standards. Major cohort studies from Sweden (13), the United States (8), and Denmark (50) corroborate these links, demonstrating that sustained exposure to PM_2.5_ and NO_2_ is associated with increased incidence of late-life depression and depressive symptoms. Specifically, the Swedish cohort reported that each 1 μg/m^3^ increase in PM_2.5_ was associated with a 55% higher risk of depression (HR = 1.55, 95% CI: 1.22–1.93), while the U.S. cohort showed significant associations for both PM_2.5_ (HR = 1.009, 95% CI: 1.000–1.018) and NO_2_ (HR = 1.006, 95% CI: 1.003–1.009). The convergence of findings across disparate pollution contexts suggests the neuropsychiatric effects of air pollution are likely ubiquitous, challenging the existence of a universally applicable safe exposure threshold.

The evidence delineates clear divergences in primary exposure sources and consequent public health priorities between urban and rural contexts. In rural settings, late-life depression risk is predominantly driven by household air pollution from solid fuel combustion. Empirical data indicate that a shift to clean energy sources can directly alleviate depressive symptoms (ATT = −3.659, CES-D reduction) (59), while continued reliance on solid fuels is robustly associated with comorbid sleep disorders and depression (OR = 2.11, 95% CI: 1.74–2.55) (22). Thus, the central public health imperative in rural areas involves catalyzing the transition to clean energy and optimizing indoor air quality through improved ventilation (27). Conversely, urban-dwelling older adults are subjected to a confluence of exposures, including traffic-related pollutants (notably NO_2_ and NO_x_) (8, 43) and dense built environments with limited green space (52). Accordingly, effective urban interventions must prioritize multisectoral strategies that combine traffic emission reduction with the evidence-based integration of green infrastructure, aiming to synergistically improve ambient air quality and foster mental health-supportive communities (28, 31). In terms of evidence strength, cohort studies from highly polluted regions (particularly China) feature large samples and long follow-ups, yielding robust effect estimates. Studies from low-pollution regions (e.g., Sweden, Ireland), despite smaller effect sizes, provide critical evidence for the “no safe threshold” hypothesis by detecting positive associations at low concentrations. Regarding urban-rural comparisons, rural evidence primarily derives from solid fuel exposure studies, while urban evidence mainly focuses on traffic-related pollution—distinct sources that inform different intervention priorities.

In summary, both the strength of the association between air pollution and late-life depression and the primary sources of exposure vary significantly depending on geographic region and urban-rural context. Understanding this spatial heterogeneity is crucial for developing context-specific environmental and public health strategies to safeguard older adults' mental health across diverse living environments. Having elucidated temporal and spatial influences, we now focus on pollutant characteristics and their independent and interactive effects.

### 3.3 Pollutant characteristics

Epidemiological data demonstrate heterogeneous associations between specific air pollutants and late-life depression, attributable to divergent exposure pathways, physicochemical characteristics, and biological modes of action. PM_2.5_, the most widely studied pollutant, shows consistent positive association with depression risk, yet heterogeneity in effect estimates suggests its neurotoxicity may be composition-dependent rather than solely mass-driven (5, 7, 33). For instance, a national cohort study reported that specific PM_2.5_ constituents—including black carbon (combustion-derived), organic matter, and secondary sulfate—were more strongly associated with depression risk than total PM_2.5_ mass (35). Specifically, black carbon (HR = 1.54, 95% CI: 1.44–1.64), organic matter (HR = 1.24, 95% CI: 1.16–1.34), and sulfate (HR = 1.25, 95% CI: 1.16–1.35) all showed significant associations. This finding regarding the heightened risk associated with specific components is reinforced by short-term exposure studies, which link organic matter and sulfate to an elevated risk of depression-related outpatient visits (organic matter: OR = 1.417, 95% CI: 1.245–1.612; sulfate: OR = 1.418, 95% CI: 1.247–1.613) (41). Together, these lines of evidence suggest that particulate matter from distinct sources and with specific chemical profiles exhibits differential neurotoxic potency.

Among gaseous pollutants, NO_2_, recognized as a typical marker of traffic sources, has been shown to be independently linked to the risk of late-life depression in several studies. For instance, one study reported that each 1 ppb increase in NO_2_ was associated with a 6% higher risk of depression (OR = 1.06, 95% CI: 1.04–1.08), and another study found a similar association (HR = 1.006, 95% CI: 1.003–1.009) (8, 34). In contrast, the effects of O_3_ exhibit time-dependent characteristics and interactions with environmental factors. For example, research has shown that medium-term average exposure to O_3_ is associated with depression risk (3-month moving average: OR = 1.17, 95% CI: 1.08–1.27), and high temperatures may potentiate this association (6).

Chronic exposure to sulfur dioxide (SO_2_) remains associated with elevated risk of depression and anxiety in older adults, even at the comparatively low ambient levels characteristic of modern cities—a finding that underscores its potential neurotoxicity (HR = 1.11, 95% CI: 1.07–1.16) (45). Separately, household air pollution generated by solid fuel (e.g., coal, wood) combustion represents a major and well-characterized depressive risk factor. Evidence from multiple geographic settings indicates that sustained reliance on solid fuels for household energy is associated with a higher likelihood of depressive symptoms among older adults (solid fuel for cooking: HR = 1.26, 95% CI: 1.13–1.40) (15–17), with sleep disturbances identified as a plausible mechanistic pathway (sleep-depression comorbidity: OR = 2.11, 95% CI: 1.74–2.55) (15, 22). A recent scoping review by Chavan et al. further contextualizes this by highlighting that indoor air pollution is a significant global public health challenge, with complex and multifarious health effects, including on mental health, particularly in low- and middle-income countries (60). Furthermore, simple behavioral modifications to improve indoor air quality, such as increasing window ventilation, have been observed to correlate with lower levels of depressive symptoms (high-frequency ventilation: OR = 0.67, 95% CI: 0.51–0.88), another study reported a similar finding (OR = 0.49, 95% CI: 0.43–0.57) (25, 26).

Real-world environmental exposure is inherently complex, involving coexisting factors that frequently interact. For example, robust evidence shows that greater residential green space can attenuate the detrimental effects of long-term particulate matter exposure on depressive symptoms, demonstrating the buffering and protective role of natural environments (lowest vs. highest NDVI quartile: OR = 3.75, 95% CI: 2.75–5.10) (28). The role of modifiable behaviors such as physical activity is more nuanced and context-dependent. In low-pollution environments, regular physical activity has a clear protective effect against depression in older adults (36, 61). However, engaging in high-intensity activity in heavily polluted settings may paradoxically strengthen the association between specific pollutants (e.g., NO_2_)and depression by increasing the effective inhaled dose, with the interaction reaching statistical significance (*p* < 0.05) (34). Notably, a systematic review confirmed that the benefits of physical activity for mental health outcomes—including depression—can be maintained even in the presence of pollution (62), suggesting that in most daily settings, the comprehensive benefits of moderate activity outweigh the marginal risks associated with background pollution (61, 62). These considerations underscore that a comprehensive assessment of environmental influences on late-life mental health must carefully account for the coexistence of multiple exposures and their potential interactive effects.

Turning to the pollutants themselves, PM_2.5_ is the most extensively studied pollutant, yet component-specific effects suggest that focusing solely on mass concentration may underestimate its neurotoxicity. NO_2_, as a traffic-related marker, has demonstrated independent effects across multiple geographic settings. Evidence for indoor solid fuel, though primarily from low- and middle-income countries, shows relatively large effect sizes with clear intervention benefits. Overall, evidence maturity varies across pollutants, underscoring the need for future research on specific components and mixed exposures.

### 3.4 Comparative synthesis

**Figure 3:**
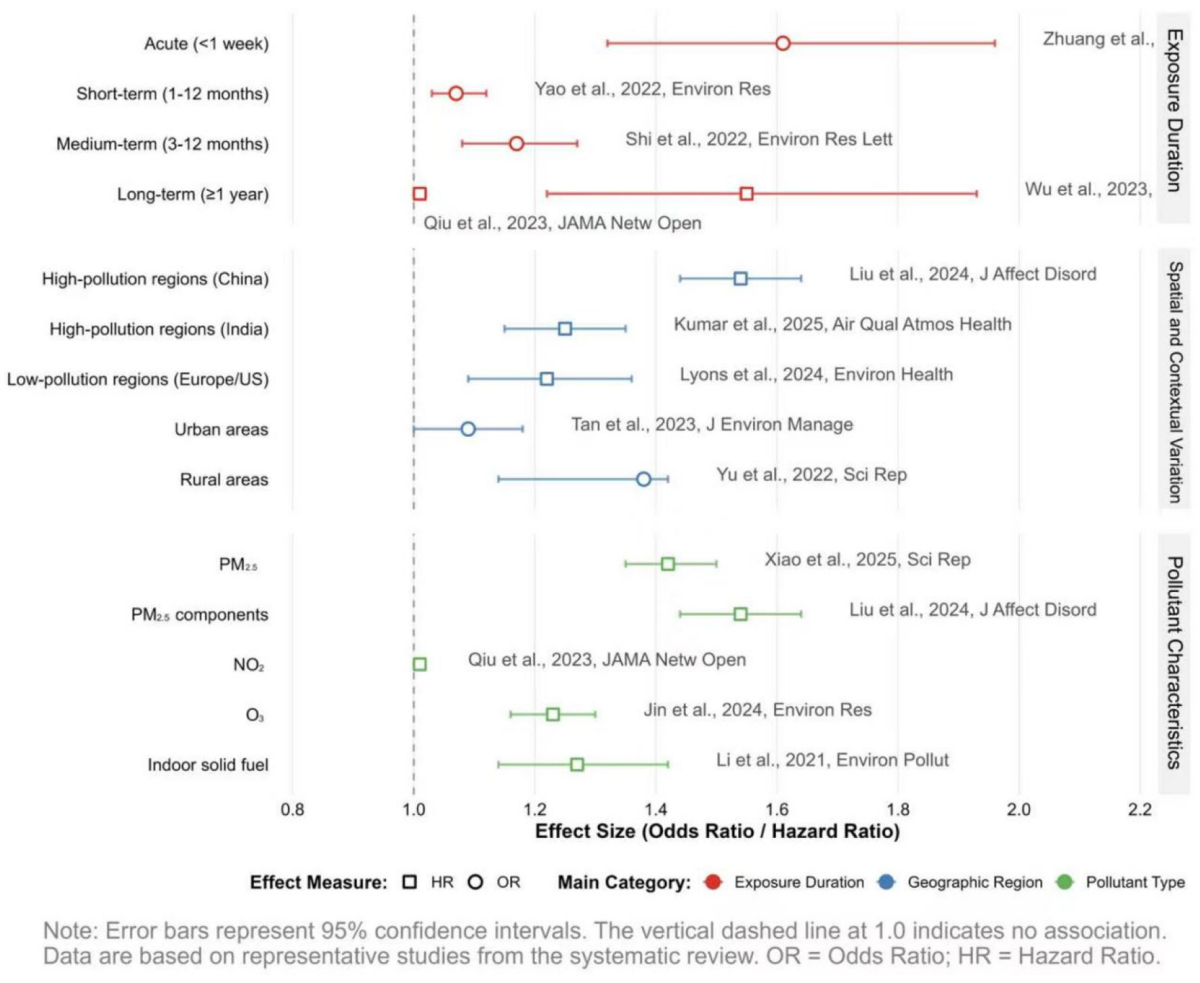
Comparison of effect estimates for the association between air pollution exposure and late-life depression risk: selection of key studies.

To facilitate a cross-dimensional comparison of association patterns and effect sizes, key epidemiological findings are visually integrated in Figure 3. Studies included in this figure were selected based on three pre-defined criteria: (1) a study design that provides strong causal inference capability (e.g., prospective cohort, case-crossover); (2) findings representative of a major exposure dimension (temporal, spatial/contextual, or pollutant-specific); and (3) the reporting of complete quantitative effect estimates (e.g., hazard ratios, odds ratios) with associated confidence intervals to enable standardized comparison. Detailed effect estimates for all 80 included studies are compiled in Appendix tables.

## 4 Discussion

### 4.1 Research progress

Accumulating epidemiological evidence demonstrates substantial heterogeneity in the effect estimates for the association between air pollution and late-life depression. This heterogeneity is structured along three principal dimensions: exposure duration, geographic and environmental context, and pollutant characteristics. Recognizing this multidimensional structure provides a crucial conceptual framework for elucidating the diverse biological mechanisms and complex environmental interactions that likely underlie these observed associations.

From a temporal perspective, the association between long-term exposure (typically defined as exceeding 1 year) and depression risk in older adults aligns with a model of cumulative biological damage, in which sustained neuroinflammation and oxidative stress are central pathophysiological pathways. Empirical evidence supports this model, including studies that have identified specific inflammatory markers (e.g., TNF-R1) as mechanistic mediators linking PM_2.5_ exposure to depression (18, 35, 45). In contrast, short-term exposure (spanning days to months) shows a stronger correlation with the acute exacerbation of depressive symptoms and increased utilization of related healthcare services (41, 57). These acute effects may be mediated by immediate psychosocial mechanisms, such as activity restriction and heightened anxiety (63).

The association between air pollution and late-life depression must be understood within the biological context of “inflammaging.” The aging process itself is characterized by elevated baseline levels of pro-inflammatory cytokines such as IL-6 and TNF-α, resulting in a persistent low-grade inflammatory state known as inflammaging. Recent studies confirm that exposure to air pollution—particularly long-term chronic exposure—can further exacerbate this inflammatory burden in older adults by activating oxidative stress pathways and the peripheral immune system (18, 64). This “dual inflammatory burden” may synergistically exceed the central nervous system's inflammatory tolerance threshold: peripheral inflammatory mediators can cross a compromised blood-brain barrier, activate microglia, and induce neuroinflammation; in turn, persistent neuroinflammation disrupts hippocampal neurogenesis and impairs synaptic plasticity—processes fundamental to emotional regulation and cognitive maintenance (65). Thus, air pollution is not merely an independent risk factor, but interacts with the biological processes of aging to jointly contribute to the development of late-life depression. The public health implication is that controlling air pollution not only improves environmental quality but also safeguards the health of vulnerable aging populations by reducing the additional inflammatory burden imposed on individuals already undergoing inflammaging.

Spatial heterogeneity in this association reflects the combined influence of local pollution concentrations, primary pollution sources, and population characteristics. Notably, a consistent positive association between long-term air pollution exposure and late-life depression risk has been observed both in highly polluted regions and in high-income countries with relatively low pollution levels, such as Sweden and Ireland (13, 14, 56). This geographical consistency reinforces air pollution's role as a adjustable risk factor for late-life depression and suggests the potential absence of an absolute safe exposure threshold. Urban-rural disparities are particularly pronounced. Among older adults in rural areas, depression risk is predominantly linked to household air pollution from solid fuel use, with sleep disturbances and exacerbated chronic conditions serving as plausible mediating pathways (15, 22, 23). Conversely, older adults in urban settings face a combination of exposures to traffic-related pollutants and the psychological stressors associated with dense built environments (8, 52). Consequently, effective prevention strategies must be tailored to account for these distinct regional and contextual characteristics.

Different pollutants exert distinct effects, a consequence of their varying sources and physicochemical characteristics. PM_2.5_, especially its combustion-derived constituents like black carbon and organic matter, shows prominent associations with depression risk in multiple studies, indicating that the chemical composition of particulate matter is a key factor in its neurotoxicity (35, 41). The independent contribution of traffic-related NO_2_ has also been robustly confirmed (8, 34). Indoor air pollution resulting from solid fuel use represents a clearly established risk factor, whereas improving indoor ventilation has been found to be associated with reduced depressive symptoms (25, 27). Furthermore, real-world exposure complexity involves coexisting factors and potential interactions. For example, residential green space has been found to buffer some of the negative mental health impacts of long-term particulate matter exposure (28). Conversely, the protective effect of physical activity against depression may be diminished or transformed into a risk factor in heavily polluted environments, likely due to increased inhalation of pollutants (34, 36). These insights underscore the need for an integrated approach when evaluating the overall environmental impact on depression in older adults. This finding is biologically significant, suggesting that air pollution-induced inflammatory burden may compound the inherent inflammatory state of aging—creating a “double-hit” for older adults and underscoring environmental regulation as a priority for safeguarding neuropsychiatric health.

In summary, the accumulated evidence supports a pathogenic model in which air pollutants contribute to the development of late-life depression through three main, interrelated pathways: promoting chronic inflammation, disrupting acute stress response regulation, and impairing health-sustaining behaviors and social engagement. The manifestation and impact of these pathways are critically modulated by the interplay of exposure duration, geographical and socioeconomic context, and pollutant characteristics. Therefore, a deep understanding of these complex interactions is essential for translating epidemiological findings into effective clinical interventions and informed public health policies.

### 4.2 Existing limitations

While accumulating evidence supports an association between air pollution and late-life depression, several persistent methodological limitations warrant careful consideration to ensure a balanced interpretation of the findings.

Exposure assessment accuracy remains a primary challenge. Most population-based studies rely on ambient concentrations from fixed-site monitors or atmospheric models, which fail to capture individual-level variations arising from daily mobility, time-activity patterns, and residential microenvironmental differences (5, 8, 43). This population-level exposure assignment can lead to non-differential misclassification of individual exposure, typically attenuating—and potentially distorting—the true effect estimates. The problem is particularly pronounced for indoor air pollution, where exposure is often inferred from self-reported solid fuel use rather than directly measured pollutant concentrations (15–17). Such indirect assessment methods inadequately reflect biologically effective doses and limit the investigation of independent effects of specific toxic constituents, such as black carbon and organic carbon.

Causal inference is further constrained by inherent design limitations of the available evidence. The body of evidence included in this review is predominantly observational, with a considerable proportion derived from cross-sectional study designs (25, 26, 33), which cannot establish temporal sequence and are susceptible to reverse causation. Although prospective cohort studies offer stronger support for temporality, they remain vulnerable to residual confounding. Factors such as socioeconomic status, individual behaviors (e.g., smoking, physical activity), and genetic predisposition may be associated with both environmental pollution exposure and depression risk independently. Even when studies adjust for known confounders, unmeasured or poorly measured variables may bias the observed associations (13, 35, 50). Evidence synthesis is further complicated by substantial methodological heterogeneity across studies. Variations in population characteristics, exposure definitions and measurements, and the criteria and instruments used to assess depressive outcomes introduce variability in effect estimates and hinder direct comparison and integration of findings (41, 57). Finally, publication bias represents an additional concern, as the academic publishing landscape may favor studies reporting statistically significant positive results, which could lead to an overestimation of the overall association strength between air pollution and late-life depression in the available literature.

Collectively, these methodological limitations must be carefully weighed when translating epidemiological evidence into public health decisions or specific intervention strategies. Future research should employ more precise individualized exposure monitoring technologies, utilize study designs that better control for confounding, and conduct more comprehensive measurement of relevant factors to provide more definitive evidence regarding the association between air pollution and late-life depression.

Furthermore, the geographical distribution of the evidence base warrants consideration. Following systematic screening of 147 records, 80 studies were included in this review. The final evidence set is predominantly derived from high-income or rapidly industrializing regions—notably China, North America, and Europe—where well-established environmental monitoring networks and long-term cohort studies enable high-quality epidemiological research. In contrast, studies from Africa and South America were notably scarce in the initial search results. This scarcity likely reflects genuine gaps in research infrastructure and data availability in these regions, rather than an artifact of our search strategy. Consequently, this geographical imbalance limits the global generalizability of our findings. Given the substantial cross-regional variations in pollution composition, population susceptibility, and socio-environmental contexts, the absence of evidence from Africa and South America represents a critical knowledge gap that warrants priority in future research.

### 4.3 Future research directions

Building upon the existing evidence, translating scientific discoveries into effective public health action and clinical practice is crucial for addressing the risk of air pollution-related late-life depression. Future work should therefore be directed toward toward constructing comprehensive strategies that integrate environmental interventions, clinical identification, and community-based support systems.

At the level of public health practice, the core task is to transform abstract environmental exposure indicators into specific, identifiable, and manageable health risks within the primary healthcare system. This implies the deliberate integration of inquiries regarding living environments into routine health assessments and chronic disease management for older adults. Specifically, environmental history-taking—encompassing factors such as household cooking fuel types (16, 17), habitual ventilation practices, and proximity to local pollution sources (25, 26)—should be routinely incorporated. In rural areas where solid fuel use remains widespread, promoting the adoption of clean energy continues to be a fundamental intervention, with evaluation studies confirming its potential benefits for mental health (27). In urban planning, proactive consideration should be given to increasing accessible green spaces in communities with high concentrations of older residents. Existing research not only corroborates the independent protective effect of green space on mood, but also suggests its potential to mitigate some of the harmful effects of air pollution (28, 42).

At the scientific and clinical translation level, future priorities should lie in deepening the understanding of underlying mechanisms and promoting precision prevention. This requires more rigorously designed studies to strengthen causal inference, for instance by utilizing large-scale policy implementations, such as “Healthy City” or “New Energy Demonstration City” initiatives, as quasi-natural experiments to evaluate their long-term mental health effects, thereby providing high-level evidence for policymaking (24, 31). Research must also better reflect complex real-world exposure scenarios, shifting from single-pollutant models to exploring mixed exposures to multiple pollutants and their interactions with other environmental stressors, such as noise and high temperature. Equally important is the need for integrative approaches combining exposomics, neuroimaging, and molecular biology to elucidate how specific pollutant components (e.g., black carbon in PM_2.5_) affect the function of neural circuits involved in emotion regulation by inducing systemic and neuroinflammation (18, 35). Clarifying these biological pathways forms the basis for discovering potential intervention targets. Advances in sensing and mobile health technologies further offer the potential to develop personalized, real-time environmental exposure monitoring and health risk early-warning systems, holding promise for providing more dynamic and individualized preventive guidance to high-risk older adults (66).

**Figure 4:**
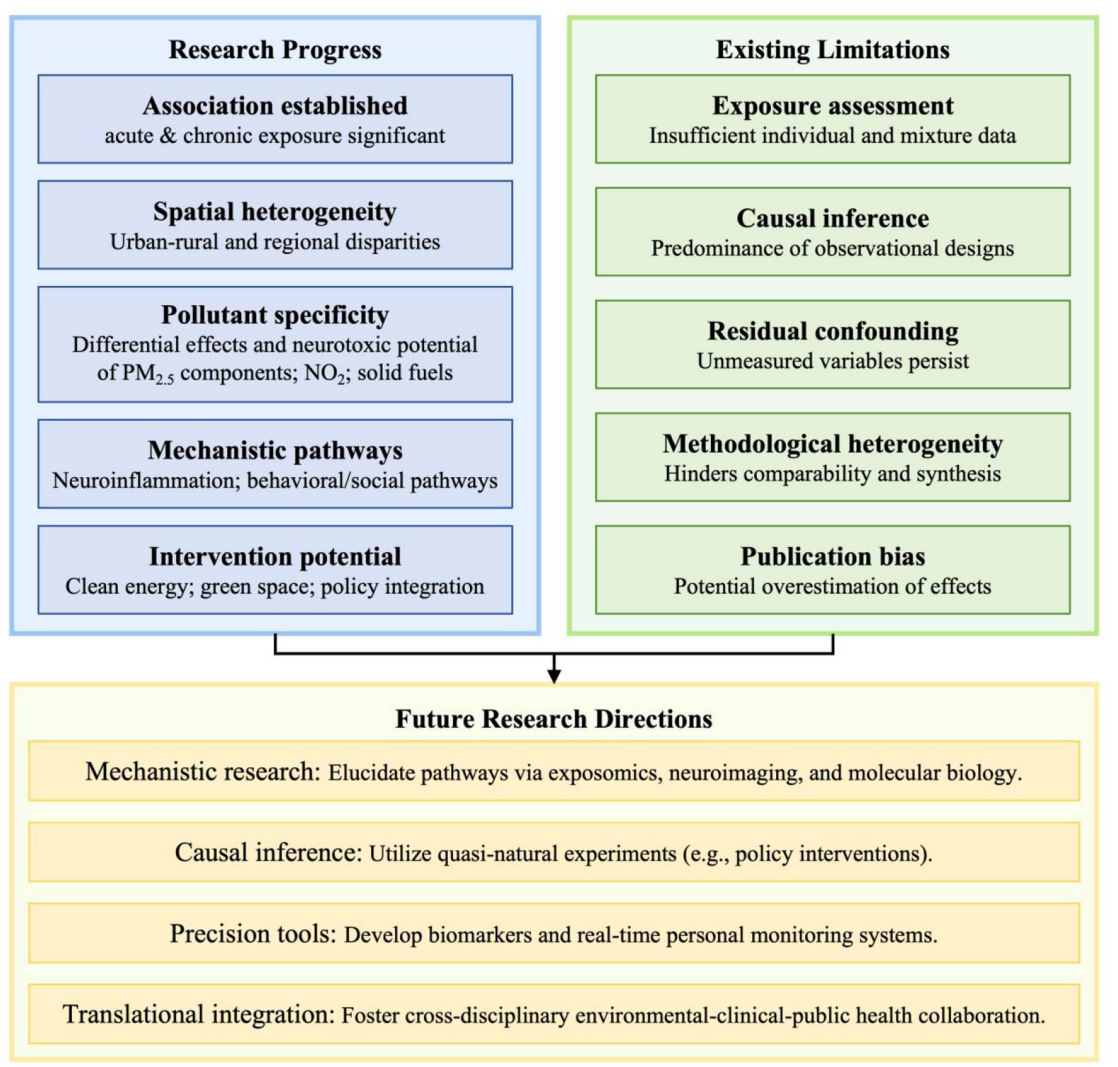
Future research directions.

In summary, addressing the threat that air pollution poses to mental health requires elevating this issue from a purely environmental concern to a core public health priority that intersects with preventive medicine, clinical mental health, and strategies for successful aging. Only by fostering deep dialogue and collaboration among environmental science, clinical medicine, and public health decision-making can we construct a multi-level intervention framework, which spanning from pollution source control to neuroprotection, to effectively safeguard the mental health and overall wellbeing of the aging population (Figure 4).

## 5 Conclusion

Based on the integration and analysis of existing epidemiological evidence, this review elucidates the complex and heterogeneous association between air pollution and the risk of late-life depression. The evidence indicates that this relationship is not uniform but exhibits distinct characteristics across the dimensions of exposure time, geographic region, and pollutant type.

From a temporal perspective, long-term chronic exposure may lead to cumulative neurological damage through processes such as sustained neuroinflammation and oxidative stress (18, 35). In contrast, short-term acute exposure is more closely associated with immediate physiological stress responses and activity restrictions (41, 57), representing two distinct yet potentially interconnected pathogenic pathways. Spatially, the association remains significant across a wide range of settings—from highly polluted regions to areas with relatively low pollution concentrations in Europe and North America—suggesting the broad scope of its impact (13, 14). Concurrently, urban-rural disparities define two primary exposure scenarios: indoor solid fuel combustion is a prominent risk factor in rural areas (16, 17), while traffic-related pollutants (e.g., NO_2_, NO_x_) exert a more substantial influence in urban environments (8, 43). Regarding pollutant characteristics, specific components of PM_2.5_–such as black carbon and organic carbon—as well as pollutants like NO_2_ demonstrate relatively clear associations with depression risk (35, 41). Additionally, interactions among environmental factors are evident; for example, green spaces have been found to mitigate some of the adverse psychological impacts of air pollution (28).

In summary, the current body of evidence indicates that air pollution is an important and modifiable environmental factor influencing late-life depression. This retrospective synthesis suggests that, against the backdrop of an increasingly aging population, air quality control strategies should be more closely aligned with public health objectives focused on promoting healthy aging, particularly the preservation of cognitive and emotional function. Future research should focus on improving exposure assessment precision, strengthening causal inference, and deepening exploration of relevant biological mechanisms. These efforts will provide a basis for developing effective comprehensive interventions tailored to different vulnerable subgroups, such as the oldest-old in rural or urban settings. Through collaboration across multiple disciplines, including environmental science, clinical medicine, and public health, it is feasible to mitigate the adverse neuropsychological effects of environmental exposures and thereby support improvements in quality of life among aging populations.
