# Supplementary material for: A multidimensional narrative review of the association between air pollution and late-life depression risk
Source: Front Public Health. 2026 Apr 22;14:1813992. doi: 10.3389/fpubh.2026.1813992 (PMC13143914; doi:10.3389/fpubh.2026.1813992)
Supplement: Supplementary file 1 [file Table_1.docx]

**Appendix**

Appendix table 1. **Literature list of short-term exposure studies in older adults**

| **Exposure** | **Citation** | **E.M.** | **Point Estimate** | **95% CI** |
| --- | --- | --- | --- | --- |
| PM_2.5_ | Association of depressive symptoms with ambient PM_2.5_... - Yao et al., 2022 | OR | 1.07 | (1.03, 1.12) |
| CO, NO_x_ | Associations Between Symptoms of Depression and Air Pollutant Exposure... - Wang et al., 2022 | OR | CO : 1.232;  NO_x_ : 1.312 | CO: (1.116, 1.361);  NO_x_: (1.158, 1.488) |
| O_3_ | Association of ambient ozone exposure with anxiety and depression... - Shi et al., 2022 | OR | Depression: 1.17;  Anxiety: 1.25 | Depression: (1.08, 1.27);  Anxiety: (1.15, 1.37) |
| PM_2.5_ | A National Study on the Impact of Wildfire Smoke... - Do et al., 2025 | Rate Ratio | 0.94 | (0.90, 0.99) |
| Noise, PM_2.5_, TVOCs, etc. | Feasibility of Deploying Home-Based Digital Technology... - Au-Yeung et al., 2024 | Spearman's ρ | Not provided | Not provided |
| PM_2.5_, Black Carbon, Ammonium, etc. | Effects of short- and long-term exposures to multiple air pollutants... - Tong et al., 2024 | β, OR | PM_2.5_: β=0.009, OR=1.005;  BC : β=0.173, OR=1.126 | PM_2.5_: β (0.003, 0.015),  OR (1.004, 1.006);  BC: β (0.007, 0.339),  OR (1.125, 1.127) |
| PM_2.5_, OM, SO4²^-^ | Short-Term Exposure toPM_2.5_ Chemical Components... - Zhuang et al., 2024 | OR | PM_2.5_: 1.607;  OM: 1.417;  SO4²^-^: 1.418 | PM_2.5_: (1.321, 1.956);  OM: (1.245, 1.612);  SO4²^-^: (1.247, 1.613) |
| NO_2_, PM_2.5_ | Disentangling impacts of multiple pollutants on acute cardiovascular events... - Humphrey et al., 2024 | Excess Risk % | 1.51% (per 10-unit NO_2_) | (1.22%, 1.80%) |
| PM_2.5_, PM_10_, SO_2_, CO | Short-term attributable risk and economic burden... - Fu et al., 2025 | OR | PM_2.5_ (per 10 μg/m³) | (1.001, 1.004) |
| O_3_, PM_2.5_ | The association between ozone and fine particles and mental health-related ED visits... - Nguyen et al., 2021 | % Change | 1.87% (O_3_, 7-day lag) | (0.62%, 3.15%) |
| PM_2.5_ | Exposure and perception of PM_2.5_ pollution on the mental stress of pregnant women - Li et al., 2021 | OR | 1.1376 | (1.0826, 1.1953) |
| Note: This table systematically compiles studies with exposure durations ranging from one day to one year, including acute exposure (less than one week). | | | | |

Appendix table 2. Literature list of long-term exposure studies **in older adults**

| **Exposure** | **Citation** | **E.M.** | **Point Estimate** | **95% CI** |
| --- | --- | --- | --- | --- |
| PM_2.5_, PM_10_, SO_2_ | Ambient air pollution and the health-related quality of life... - Tan et al., 2023 | β | See dimension-specific analysis | Not provided per dimension |
| Air pollution (satisfaction) | Multidimensional health heterogeneity of Chinese older adults... - Hu et al., 2023 | OR | e.g., 0.457 | e.g., (0.269, 0.779) |
| PM_2.5_, PM_10_, Greenness | Association between air pollution (PM_10_, PM_2.5_), greenness and depression... - Park et al., 2024 | OR | PM_2.5_ (NDVI Q1): 3.75;  PM_10_ (NDVI Q1): 1.88 | PM_2.5_: (2.75, 5.10);  PM_10_: (1.58, 2.25) |
| PM_2.5_, PM_10_, Noise | The impact of healthy community environment and sleep quality... - Wei et al., 2025 | β | Health env. → Sleep quality: β=0.133 | Not provided |
| Composite index (AQI/API) | The impact of new energy demonstration city policy... - Wan et al., 2025 | β | -0.1580  (policy dummy) | Not provided (p < 0.1) |
| PM_2.5_, PM_10_ | Fine particulate matter, vitamin D, physical activity, and major depressive disorder... - Wu et al., 2022 | OR | 1.096  (Q4 vs. Q1 for PM_2.5_) | (1.023, 1.175) |
| Drinking water quality | Associations among drinking water quality, dyslipidemia, and cognitive function... - Pan et al., 2022 | β | Global cognition: 0.58 | Not provided |
| NO_2_, PM_2.5_ | Physical Activity-Induced Modification of the Association... - Park et al., 2024 | OR | 1.06 (per 1 ppb NO_2_) | (1.04, 1.08) |
| PM_2.5_, PM_10_, NO_x_ | Air pollution, social engagement, and depression in older adults... - Wu et al., 2023 | HR | PM_2.5_: 1.55  (per 1 μg/m³) | (1.22, 1.93) |
| PM_2.5_, PM_10_, SO_2_ | Ambient air pollution and the health-related quality of life... - Tan et al., 2023 | β | PM_2.5_ on Anxiety/Depression: 0.006* | (SE: 0.003) |
| Solid fuel | Effects of long-term household air pollution exposure from solid fuel use... - Li et al., 2021 | HR | Heating: 1.27;  Cooking: 1.26 | Heating: (1.14, 1.42);  Cooking: (1.13, 1.40) |
| PM_2.5_, SO_2_ | The impact of environmental pollution on the physical health... - Fan et al., 2022 | dy/ dx | Not directly given | Not provided |
| O_3_, NO_2_, PM_2.5_ | Association of Long-term Exposure to Air Pollution With Late-Life Depression... - Qiu et al., 2023 | HR | PM_2.5_: 1.009;  NO_2_: 1.006;  O_3_: 1.021 | PM_2.5_: (1.000, 1.018);  NO_2_: (1.003, 1.009);  O_3_: (1.016, 1.026) |
| PM_2.5_, TNF-R1 | The increased risk of exposure to fine particulate matter for depression incidence... - Chang et al., 2025 | HR | 1.175 (PM_2.5_ per IQR) | (1.012, 1.363) |
| PM_2.5_, Solid fuel | Industrialization, indoor and ambient air quality, and elderly mental health - Ao et al., 2021 | β, OR | PM_2.5_: β=0.0237  (CES-D score) | SE: 0.0102 |
| Solid fuel | Mediating Factors Explaining the Associations between Solid Fuel Use... - Yu et al., 2022 | β | -0.045 | SE: 0.007 |
| PM_2.5_, PM_10_, SO_2_, CO, O_3_ | Long-term effects of common air pollutants and depression... - Xiao et al., 2025 | HR | PM_2.5_: 1.42;  O_3_: 0.59 | PM_2.5_: (1.35, 1.50);  O_3_: (0.57, 0.61) |
| Green space | “Your soul will rest in the fresh air”... - Hawkins et al., 2025 | N/A | N/A | N/A |
| O_3_, SO_2_, NO_2_ | Activity-related dyspnea in older adults... - Verschoor et al., 2022 | OR | Depression only: 3.68 | (3.15, 4.29) |
| Coal power plant emissions | Effect of residential proximity to the lignite-fired power plant... - Mata et al., 2022 | Regression Coef. | 1.003  (<10km vs >15km, crude) | (-0.313, 2.319) |
| PM_2.5_ components (BC, OM, SO4²^-^) | Long-term effects of fine particulate matter components on depression... - Liu et al., 2024 | HR | BC: 1.54;  OM: 1.24 | BC: (1.44, 1.64);  OM: (1.16, 1.34) |
| PM_2.5_ | Fine Particulate Matter Is Associated With Lower Executive Functioning... - Grineski et al., 2025 | Not reported | Not reported | Not reported |
| PM_2.5_, PM_10_ | Association between ambient particulate matters and anhedonia... - Xie et al., 2024 | β | RSAS (PM_2.5_): 1.238;  RPAS (PM_2.5_): 1.888 | RSAS (PM_2.5_): (0.353, 2.123);  RPAS (PM_2.5_): (0.699, 3.078) |
| O_3_, PM_2.5_ | Long-term ambient ozone, omega-3 fatty acid... - Jin et al., 2024 | HR | Depression: 1.231;  Anxiety: 1.237 | Depression: (1.163, 1.303);  Anxiety: (1.168, 1.310) |
| PM_2.5_ | The independent role of fine particulate matter and genetic liability... - Liao et al., 2025 | β | -0.083 (on MMSE) | (-0.0973, -0.0688) |
| PM_2.5_, Heat stress | Mental health effects of urban landscape and environmental stress... - Wang et al., 2025 | Relative Contribution % | PM_2.5_ contribution to GDS: 12.29% | Not provided |
| PM_2.5_, Green space | China’s Healthy City Pilot Policy Improves Physical and Mental Health... - Zhang et al., 2025 | β | -0.957 (on CES-D) | Not provided |
| PM_2.5_, Green space | The mitigation effects of residential green space and low air pollution... - Wu et al., 2025 | HR | 2.44 (combined effect) | (2.21, 2.71) |
| PM_2.5_ | Association Between Ambient Fine Particulate Matter and Physical Functioning... - Wang et al., 2022 | β, OR | Grip strength:  -220g /60kg | (127, 312)g |
| SO_2_, AQI | Correlation between air pollution and cognitive impairment... - Liu et al., 2023 | OR | 1.4633 (per unit AQI) | (1.20899, 1.77116) |
| PM_2.5_ components | Cognitive impairment associated with individual and joint exposure... - Deng et al., 2024 | β | Range: -1.477 (Nitrate) to -0.822 (BC) | See原文表1及图1 |
| Green space | Associations between neighborhood park access and longitudinal change... - Besser et al., 2021 | OR | 1.04 (per 0.10 increase in park proportion) | (1.00, 1.08) |
| Noise | Nonlinear effect of urban noise pollution on depression... - Jin et al., 2024 | β | Range: 0.1201 (0.25 quantile) to 0.5709 (0.85 quantile) | See文中表1 |
| PM_2.5_, PM_10_, SO_2_, O_3_ | Impact of exposure to ambient air pollution on health related quality of life... - Xiao et al., 2025 | Not provided | Not provided | Not provided |
| PM_2.5_, Solid fuel | The impact of living environmental factors on cognitive function... - Luo et al., 2024 | HR | See tables | See tables |
| PM_1_, PM_2.5_, PM_10_, O_3_, NO_2_ | Physical activity modifies the association between ambient air pollution... - Chen et al., 2025 | aHR | Multiple values,  see article | Multiple values, see article |
| PM_2.5_, Solid fuel, other | Environmental determinants of depression risk in China... - Xie et al., 2025 | OR, HR | Multiple values,  see article | Multiple values, see article |
| SO_2_ | Long-term exposure to low-concentration sulfur dioxide and mental disorders... - Yuan et al., 2025 | HR, OR | Depression: HR 1.11 | (1.07, 1.16) |
| PM_2.5_, NO_2_ | Outdoor Air Pollution Exposure and Inter-relation... - Petkus et al., 2021 | β | PM_2.5_: β = -0.144;  NO_2_: β = -0.157 | PM_2.5_: (-0.261, -0.028);  NO_2_: (-0.291, -0.022) |
| Pollution perception | The impact of environmental pollution on aging anxiety... - Zhang, 2025 | Unstd. Coef. | Total effect: B=0.120 | [0.038, 0.202] |
| PM_2.5_, NO_2_, Greenness | Associations of neighbourhood environmental attributes and socio-economic status... - Barnett et al., 2023 | Regression Coef. | -0.218 (PM_2.5_ on PCS) | (-0.393, -0.044) |
| Solid fuel | The effect of clean cooking on the social participation... - Li et al., 2024 | HR | 1.31  (clean vs. solid fuel) | (1.19, 1.44) |
| Solid fuel | Ecological Sustainability and Households’ Wellbeing... - Zhang et al., 2022 | ATT | -3.659 (score reduction) | Not provided |
| Solid fuel | Solid fuels use for cooking and sleep health... - Yu et al., 2021 | OR | 2.11 | (1.74, 2.55) |
| Vision/hearing impairment | Association between vision and hearing impairment and successful aging... - Gopinath et al., 2021 | OR | Not provided | Not provided |
| PM_2.5_ | Long-term exposure to PM_2.5_ air pollution and mental health... - Lyons et al., 2024 | β, OR | CES-D: β=0.0312;  Anxiety :OR=1.217 | CES-D: (0.0093, 0.0531);  Anxiety: (1.085, 1.364) |
| PM_2.5_ | Air pollution and mental health... - Chen et al., 2023 | β | -0.243 (per μg/m³) | SE: 0.059 |
| Solid fuel | Does clean cooking energy improve mental health?... - Liu et al., 2022 | DID Coef. | -1.522 (CES-D index) | Not reported |
| PM_2.5_, PM_10_, NO_2_, Mn | Cohort profile: the Environmental-Pollution-Induced Neurological Effects... - Jang et al., 2021 | Not reported | Not reported | Not reported |
| PM_2.5_, NO_2_, BC | Long-term exposure to air pollution and risk of dementia... - Andersen et al., 2025 | HR | NO_2_: 1.25;  PM_2.5_: 1.14 | NO_2_: (1.22, 1.28);  PM_2.5_: (1.12, 1.16) |
| PM_2.5_ | Railways to better minds... - Zong et al., 2025 | Indirect Effect | 0.038 | (0.026, 0.050) |
| AQI/API, PM_2.5_ | Population Health Effects of Air Pollution... - Shen et al., 2021 | β | -0.1512 | Not provided |
| PM_2.5_, PM_10_, NO_2_, SO_2_, CO, Solid fuel | Assessing physical and mental health impacts of solid cooking fuel emissions - Kumar et al., 2025 | Cohen's d (range) | Wide range (e.g., PM_2.5_: -8,282.56 to 4,860.98) | Wide range |
| PM_2.5_, Green space | Longitudinal mental health associations of relocation... - Sui et al., 2025 | β | -3.869 | [-7.583, -0.155] |
| PM_2.5_, PM_10_, NO_2_, SO_2_, O_3_ | Exposures to ambient air pollutants increase prevalence of sleep disorder... - Zhou et al., 2023 | OR | PM_2.5_: 1.089;  PM_10_: 1.147 | PM_2.5_: (1.003, 1.182);  PM_10_: (1.062, 1.240) |
| Note: This table systematically compiles studies with exposure durations of one year or more (≥1 year), including investigations of long-term, cumulative, or multi-year average exposure. | | | | |

Appendix table 3. Literature list of high-pollution area studies **in older adults**

| **Exposure** | **Citation** | **E.M.** | **Point Estimate** | **95% CI** |
| --- | --- | --- | --- | --- |
| PM_2.5_ | Association of depressive symptoms with ambient PM_2.5_... - Yao et al., 2022 | OR | 1.07 (180-day window) | (1.03, 1.12) |
| Green space | Impact of green infrastructure in smart older adult care communities... - Wang et al., 2025 | Regression Coef. | -0.14  (example for green coverage vs. SDS) | Not provided |
| Solid fuel | The mediating effect of sleep quality on solid cooking fuel use... - Luo et al., 2024 | OR | 1.38 | (1.12, 1.70) |
| PM_2.5_, PM_10_, SO_2_ | Ambient air pollution and the health-related quality of life... - Tan et al., 2023 | β | See dimension-specific analysis | Not provided per dimension |
| Air pollution (satisfaction) | Multidimensional health heterogeneity of Chinese older adults... - Hu et al., 2023 | OR | e.g., 0.457 (satisfied with air quality) | e.g., (0.269, 0.779) |
| CO, NO_x_ | Associations Between Symptoms of Depression and Air Pollutant Exposure... - Wang et al., 2022 | OR | CO (7-day): 1.232 | CO: (1.116, 1.361) |
| PM_2.5_, Solid fuel | The impact of new energy demonstration city policy... - Wan et al., 2025 | β | -0.1580  (policy dummy) | Not provided (p < 0.1) |
| Health insurance (PM_2.5_ controlled) | The contribution of Urban and Rural Resident Basic Medical Insurance... - Sun et al., 2021 | Concentration Index | -0.1067  (depression incidence) | N/A |
| Drinking water quality | Associations among drinking water quality, dyslipidemia, and cognitive function... - Pan et al., 2022 | β | Global cognition: 0.58 | Not provided |
| Indoor ventilation frequency | Association between window ventilation frequency and depressive symptoms... - Luo et al., 2025 | OR | 0.67 | (0.51, 0.88) |
| PM_2.5_, PM_10_, SO_2_ | Ambient air pollution and the health-related quality of life... - Tan et al., 2023 | β | PM_2.5_ on Anxiety/Depression: 0.006* | (SE: 0.003) |
| Solid fuel | Effects of long-term household air pollution exposure from solid fuel use... - Li et al., 2021 | HR | Heating: 1.27 | (1.14, 1.42) |
| O_3_ | Association of ambient ozone exposure with anxiety and depression... - Shi et al., 2022 | OR | Depression: 1.17 | (1.08, 1.27) |
| Air satisfaction | Multidimensional health heterogeneity of Chinese older adults... - Hu et al., 2023 | OR | Not provided | Not provided |
| PM_2.5_, SO_2_, Wastewater | The impact of environmental pollution on the physical health... - Fan et al., 2022 | Marginal Effect | Not directly given | Not provided |
| Indoor ventilation, Solid fuel | Associations of Indoor Ventilation Frequency with Depression and Anxiety... - Du et al., 2024 | OR | Depression: 0.49 | (0.43, 0.57) |
| PM_2.5_, Solid fuel | Industrialization, indoor and ambient air quality, and elderly mental health - Ao et al., 2021 | β, OR | PM_2.5_: β=0.0237 (CES-D score) | SE: 0.0102 |
| Solid fuel | Mediating Factors Explaining the Associations between Solid Fuel Use... - Yu et al., 2022 | β | -0.045 (depression as mediator) | SE: 0.007 |
| PM_2.5_, PM_10_, SO_2_, CO, O_3_ | Long-term effects of common air pollutants and depression... - Xiao et al., 2025 | HR | PM_2.5_: 1.42 | (1.35, 1.50) |
| Coal power plant emissions | Effect of residential proximity to the lignite-fired power plant... - Mata et al., 2022 | Regression Coef. | 1.003 (<10km vs >15km, crude) | (-0.313, 2.319) |
| PM_2.5_ components | Long-term effects of fine particulate matter components on depression... - Liu et al., 2024 | HR | BC: 1.54 | (1.44, 1.64) |
| Drinking water quality | Associations among drinking water quality, dyslipidemia, and cognitive function... - Pan et al., 2022 | β | Global cognition: 0.58 | Not provided |
| PM_2.5_, PM_10_ | Association between ambient particulate matters and anhedonia... - Xie et al., 2024 | β | RSAS (PM_2.5_): 1.238 | (0.353, 2.123) |
| PM_2.5_, O_3_, Components | Effects of short- and long-term exposures to multiple air pollutants... - Tong et al., 2024 | β, OR | PM_2.5_ (2-year): β=0.009 | β: (0.003, 0.015) |
| PM_2.5_ | The independent role of fine particulate matter and genetic liability... - Liao et al., 2025 | β | -0.083 (on MMSE) | (-0.0973, -0.0688) |
| PM_2.5_, Heat stress | Mental health effects of urban landscape and environmental stress... - Wang et al., 2025 | Relative Contribution % | PM_2.5_ contribution to GDS: 12.29% | Not provided |
| PM_2.5_, Green space | China’s Healthy City Pilot Policy Improves Physical and Mental Health... - Zhang et al., 2025 | β | -0.957 (on CES-D) | Not provided |
| Asthma (India) | Breathlessness and Beyond... - Adeagbo et al., 2025 | AOR | Physician-diagnosed asthma & depression: 2.10 | (1.41, 3.14) |
| PM_2.5_, OM, SO4²^-^ | Short-Term Exposure to PM_2.5_ Chemical Components... - Zhuang et al., 2024 | OR | PM_2.5_: 1.607 | (1.321, 1.956) |
| CHDI (incl. depression) | Spatial variations and influencing factors of Cumulative Health Deficit Index... - Xiang et al., 2023 | No specific effect value | N/A | N/A |
| Community environment perception | Community Environment Perception on Depression... - Zhang et al., 2021 | β | Total community env. perception: β = 0.532 | (0.462, 0.602) |
| Environmental pollution perception | The impact of environmental pollution on aging anxiety... - Zhang, 2025 | Unstd. Coef. | Total effect: B=0.120 | [0.038, 0.202] |
| PM1, PM_2.5_, PM_10_, O_3_, NO_2_ | Physical activity modifies the association between ambient air pollution... - Chen et al., 2025 | aHR | Multiple values | Multiple values |
| PM_2.5_, Solid fuel, other | Environmental determinants of depression risk in China... - Xie et al., 2025 | OR, HR | Multiple values | Multiple values |
| Solid fuel | The effect of clean cooking on the social participation... - Li et al., 2024 | HR | 1.31 (clean vs. solid fuel) | (1.19, 1.44) |
| Solid fuel | Ecological Sustainability and Households’ Wellbeing... - Zhang et al., 2022 | ATT | -3.659 (score reduction) | Not provided |
| Solid fuel | Solid fuels use for cooking and sleep health... - Yu et al., 2021 | OR | 2.11 (sleep problems & depression co-occurrence) | (1.74, 2.55) |
| PM_2.5_ | Air pollution and mental health... - Chen et al., 2023 | β | -0.243 (per μg/m³) | SE: 0.059 |
| Solid fuel | Does clean cooking energy improve mental health?... - Liu et al., 2022 | DID Coef. | -1.522 (CES-D index) | Not reported |
| PM_2.5_ | Railways to better minds... - Zong et al., 2025 | Indirect Effect | 0.038 | [0.026, 0.050] |
| AQI/API, PM_2.5_ | Population Health Effects of Air Pollution... - Shen et al., 2021 | β | -0.1512 (AQI on mental health) | Not provided |
| PM_2.5_, PM_10_, NO_2_NO_2_, SO_2_, CO, Solid fuel | Assessing physical and mental health impacts of solid cooking fuel emissions - Kumar et al., 2025 | Cohen's d (range) | Wide range | Wide range |
| PM_2.5_, PM_10_, NO_2_, SO_2_, O_3_ | Exposures to ambient air pollutants increase prevalence of sleep disorder... - Zhou et al., 2023 | OR | PM_2.5_: 1.089 | (1.003, 1.182) |
| PM_2.5_ | Exposure and perception of PM_2.5_ pollution on the mental stress of pregnant women - Li et al., 2021 | OR | 1.1376 (per unit ADD increase) | (1.0826, 1.1953) |
| Note: Primarily includes studies conducted in regions with historically high air pollution levels, such as China, India, and other rapidly industrializing countries. | | | | |

Appendix table 4. Literature list of low-pollution area studies **in older adults**

| **Exposure** | **Citation** | **E.M.** | **Point Estimate** | **95% CI** |
| --- | --- | --- | --- | --- |
| PM_2.5_, PM_10_ | Fine particulate matter, vitamin D, physical activity, and major depressive disorder... - Wu et al., 2022 | OR | 1.096 (Q4 vs. Q1 for PM_2.5_) | (1.023, 1.175) |
| PM_2.5_, PM_10_, NO_x_ | Air pollution, social engagement, and depression in older adults... - Wu et al., 2023 | HR | PM_2.5_: 1.55 (per 1 μg/m³) | (1.22, 1.93) |
| PM_2.5_, NO_2_, O_3_ | Association of Long-term Exposure to Air Pollution With Late-Life Depression... - Qiu et al., 2023 | HR | PM_2.5_: 1.009 | (1.000, 1.018) |
| O_3_, SO_2_, NO_2_ | Activity-related dyspnea in older adults... - Verschoor et al., 2022 | OR | Depression only: 3.68 | (3.15, 4.29) |
| O_3_, PM_2.5_ | Long-term ambient ozone, omega-3 fatty acid... - Jin et al., 2024 | HR | Depression: 1.231 | (1.163, 1.303) |
| PM_2.5_, Green space | The mitigation effects of residential green space and low air pollution... - Wu et al., 2025 | HR | 2.44 (combined effect) | (2.21, 2.71) |
| Green space (Park access) | Associations between neighborhood park access and longitudinal change... - Besser et al., 2021 | OR | 1.04 (per 0.10 increase in park proportion) | (1.00, 1.08) |
| SO_2_ (low concentration) | Long-term exposure to low-concentration sulfur dioxide and mental disorders... - Yuan et al., 2025 | HR | Depression: HR 1.11 (continuous) | (1.07, 1.16) |
| PM_2.5_, NO_2_ | Outdoor Air Pollution Exposure and Inter-relation... - Petkus et al., 2021 | β | PM_2.5_: β = -0.144 | (-0.261, -0.028) |
| PM_2.5_, NO_2_, Greenness | Associations of neighbourhood environmental attributes and socio-economic status... - Barnett et al., 2023 | Regression Coef. (b) | -0.218 (PM_2.5_ on PCS) | (-0.393, -0.044) |
| Vision/hearing impairment | Association between vision and hearing impairment and successful aging... - Gopinath et al., 2021 | OR | Not provided for depression alone | Not provided |
| PM_2.5_ | Long-term exposure to PM_2.5_ air pollution and mental health... - Lyons et al., 2024 | β, OR | CES-D: β=0.0312 | (0.0093, 0.0531) |
| NO_2_, PM_2.5_, SO_2_, O_3_ | Disentangling impacts of multiple pollutants on acute cardiovascular events... - Humphrey et al., 2024 | Excess Risk % | 1.51%  (per 10-unit NO_2_) | (1.22%, 1.80%) |
| PM_2.5_, PM_10_, NO_2_, Mn | Cohort profile: the Environmental-Pollution-Induced Neurological Effects... - Jang et al., 2021 | Not reported | Not reported | Not reported |
| PM_2.5_, NO_2_, BC | Long-term exposure to air pollution and risk of dementia... - Andersen et al., 2025 | HR | NO_2_: 1.25 | (1.22, 1.28) |
| O_3_, PM_2.5_ | The association between ozone and fine particles and mental health-related ED visits... - Nguyen et al., 2021 | % Change | 1.87% (O_3_, 7-day lag) | (0.62%, 3.15%) |
| Wildfire smoke (Qualitative) | What can we do when the smoke rolls in?... - Humphreys et al., 2022 | N/A (Qualitative) | N/A | N/A |
| Note: Primarily includes studies conducted in regions with relatively low air pollution levels, such as Europe, North America, and other developed countries. | | | | |

Appendix table 5. Literature list of urban environment studies **in older adults**

| **Exposure** | **Citation** | **E.M.** | **Point Estimate** | **95% CI** |
| --- | --- | --- | --- | --- |
| PM_2.5_, PM_10_, SO_2_ | Ambient air pollution and the health-related quality of life... - Tan et al., 2023 | β | See dimension-specific analysis | Not provided per dimension |
| Green space | Association between air pollution (PM_10_, PM_2.5_), greenness and depression... - Park et al., 2024 | OR | PM_2.5_ (NDVI Q1): 3.75 | (2.75, 5.10) |
| PM_2.5_, PM_10_, Noise | The impact of healthy community environment and sleep quality... - Wei et al., 2025 | β | Health env. → Sleep quality: β=0.133 | Not provided (p-values) |
| Composite index (AQI/API) | The impact of new energy demonstration city policy... - Wan et al., 2025 | β | -0.1580  (policy dummy) | Not provided (p < 0.1) |
| NO_2_, PM_2.5_ | Physical Activity-Induced Modification of the Association... - Park et al., 2024 | OR | 1.06 (per 1 ppb NO_2_) | (1.04, 1.08) |
| PM_2.5_, PM_10_, SO_2_ | Ambient air pollution and the health-related quality of life... - Tan et al., 2023 | β | PM_2.5_ on Anxiety/Depression: 0.006* | (SE: 0.003) |
| Indoor ventilation,  Solid fuel | Associations of Indoor Ventilation Frequency with Depression and Anxiety... - Du et al., 2024 | OR | Depression (high freq.): 0.49 | (0.43, 0.57) |
| PM_2.5_, NO_2_, O_3_ | Association of Long-term Exposure to Air Pollution With Late-Life Depression... - Qiu et al., 2023 | HR | PM_2.5_: 1.009 | (1.000, 1.018) |
| PM_2.5_, TNF-R1 (mediator) | The increased risk of exposure to fine particulate matter for depression incidence... - Chang et al., 2025 | HR | 1.175 (PM_2.5_ per IQR) | (1.012, 1.363) |
| PM_2.5_, Solid fuel | Industrialization, indoor and ambient air quality, and elderly mental health - Ao et al., 2021 | β, OR | PM_2.5_: β=0.0237 (CES-D score) | SE: 0.0102 |
| Hearing impairment (Singapore) | The Impact of Hearing Impairment on Health Indicators... - Fenwick et al., 2023 | OR | e.g., Unilateral mild HI: 4.18  (ns after correction) | (0.85, 20.56) |
| PM_2.5_, Heat stress | Mental health effects of urban landscape and environmental stress... - Wang et al., 2025 | Relative Contribution % | PM_2.5_ contribution to GDS: 12.29% | Not provided |
| PM_2.5_, Green space | China’s Healthy City Pilot Policy Improves Physical and Mental Health... - Zhang et al., 2025 | Beta | -0.957 (on CES-D) | Not provided |
| Noise | Nonlinear effect of urban noise pollution on depression... - Jin et al., 2024 | β | Range: 0.1201 to 0.5709 | See文中表1 |
| SO_2_ (low concentration) | Long-term exposure to low-concentration sulfur dioxide and mental disorders... - Yuan et al., 2025 | HR, OR | Depression: HR 1.11 (continuous) | (1.07, 1.16) |
| Community environment perception | Community Environment Perception on Depression... - Zhang et al., 2021 | β | Total community env. perception: β = 0.532 | (0.462, 0.602) |
| PM_2.5_, NO_2_, Greenness | Associations of neighbourhood environmental attributes and socio-economic status... - Barnett et al., 2023 | Regression Coef. | -0.218 (PM_2.5_ on PCS) | (-0.393, -0.044) |
| NO_2_, PM_2.5_, SO_2_, O_3_ | Disentangling impacts of multiple pollutants on acute cardiovascular events... - Humphrey et al., 2024 | Excess Risk % | 1.51%  (per 10-unit NO_2_) | (1.22%, 1.80%) |
| PM_2.5_, PM_10_, NO_2_, SO_2_, O_3_ | Exposures to ambient air pollutants increase prevalence of sleep disorder... - Zhou et al., 2023 | OR | PM_2.5_: 1.089 | (1.003, 1.182) |
| Note: Studies explicitly focused on urban settings or with a majority urban sample. | | | | |

Appendix table 6. Literature list of rural environment studies **in older adults**

| **Exposure** | **Citation** | **E.M.** | **Point Estimate** | **95% CI** |
| --- | --- | --- | --- | --- |
| Solid fuel | The mediating effect of sleep quality on solid cooking fuel use... - Luo et al., 2024 | OR | 1.38 | (1.12, 1.70) |
| PM_2.5_, PM_10_, SO_2_ | Impact of exposure to ambient air pollution on health related quality of life... - Xiao et al., 2025 | Not provided | Not provided | Not provided |
| Solid fuel | Ecological Sustainability and Households’ Wellbeing... - Zhang et al., 2022 | ATT | -3.659 (score reduction) | Not provided |
| Wildfire smoke (Qualitative) | What can we do when the smoke rolls in?... - Humphreys et al., 2022 | N/A (Qualitative) | N/A | N/A |
| Note: Studies explicitly focused on rural settings or with a majority rural sample. | | | | |

Appendix table 7. Literature list of overall PM_2.5_ studies **in older adults**

| **Exposure** | **Citation** | **E.M.** | **Point Estimate** | **95% CI** |
| --- | --- | --- | --- | --- |
| PM_2.5_ | Association of depressive symptoms with ambient PM_2.5_... - Yao et al., 2022 | OR | 1.07 (180-day window) | (1.03, 1.12) |
| PM_2.5_ | Ambient air pollution and the health-related quality of life... - Tan et al., 2023 | β | See dimension-specific analysis | Not provided per dimension |
| PM_2.5_ | Association between air pollution (PM_10_, PM_2.5_), greenness and depression... - Park et al., 2024 | OR | PM_2.5_ (NDVI Q1): 3.75 | (2.75, 5.10) |
| PM_2.5_ | The impact of healthy community environment and sleep quality... - Wei et al., 2025 | β | Health env. → Sleep quality: β=0.133 | Not provided (p-values) |
| PM_2.5_ | Fine particulate matter, vitamin D, physical activity, and major depressive disorder... - Wu et al., 2022 | OR | 1.096 (Q4 vs. Q1) | (1.023, 1.175) |
| PM_2.5_ | The contribution of Urban and Rural Resident Basic Medical Insurance... - Sun et al., 2021 | Concentration Index | -0.1067 (depression incidence) | N/A |
| PM_2.5_ | Physical Activity-Induced Modification of the Association... - Park et al., 2024 | OR | Not the primary exposure (NO2 was) | See NO_2_ row |
| PM_2.5_ | Air pollution, social engagement, and depression in older adults... - Wu et al., 2023 | HR | 1.55 (per 1 μg/m³) | (1.22, 1.93) |
| PM_2.5_ | Ambient air pollution and the health-related quality of life... - Tan et al., 2023 | β | PM_2.5_ on Anxiety/Depression: 0.006* | (SE: 0.003) |
| PM_2.5_ | The impact of environmental pollution on the physical health... - Fan et al., 2022 | Marginal Effect | Not directly given | Not provided |
| PM_2.5_ | Association of Long-term Exposure to Air Pollution With Late-Life Depression... - Qiu et al., 2023 | HR | 1.009 | (1.000, 1.018) |
| PM_2.5_ | The increased risk of exposure to fine particulate matter for depression incidence... - Chang et al., 2025 | HR | 1.175 (per IQR) | (1.012, 1.363) |
| PM_2.5_ | Industrialization, indoor and ambient air quality, and elderly mental health - Ao et al., 2021 | β, OR | β=0.0237  (CES-D score) | SE: 0.0102 |
| PM_2.5_ | Long-term effects of common air pollutants and depression... - Xiao et al., 2025 | HR | 1.42 | (1.35, 1.50) |
| PM_2.5_ | A National Study on the Impact of Wildfire Smoke... - Do et al., 2025 | RR | 0.94 | (0.90, 0.99) |
| PM_2.5_ | Long-term effects of fine particulate matter components on depression... - Liu et al., 2024 | HR | Reported for components, not total PM_2.5_ | See component rows |
| PM_2.5_ | Fine Particulate Matter Is Associated With Lower Executive Functioning... - Grineski et al., 2025 | Not reported | Not reported | Not reported |
| PM_2.5_ | Feasibility of Deploying Home-Based Digital Technology... - Au-Yeung et al., 2024 | Spearman's ρ | Trends reported | Not provided |
| PM_2.5_ | Association between ambient particulate matters and anhedonia... - Xie et al., 2024 | β | RSAS (PM_2.5_): 1.238 | (0.353, 2.123) |
| PM_2.5_ | Effects of short- and long-term exposures to multiple air pollutants... - Tong et al., 2024 | β, OR | PM_2.5_ (2-year): β=0.009, OR=1.005 | β: (0.003, 0.015); OR: (1.004, 1.006) |
| PM_2.5_ | Long-term ambient ozone, omega-3 fatty acid... - Jin et al., 2024 | HR | Not the primary exposure (O_3_ was) | See O_3_ row |
| PM_2.5_ | The independent role of fine particulate matter and genetic liability... - Liao et al., 2025 | β | -0.083 (on MMSE) | (-0.0973, -0.0688) |
| PM_2.5_ | Mental health effects of urban landscape and environmental stress... - Wang et al., 2025 | Relative Contribution % | 12.29% (to GDS) | Not provided |
| PM_2.5_ | China’s Healthy City Pilot Policy Improves Physical and Mental Health... - Zhang et al., 2025 | β | -0.957 (on CES-D) | Not provided |
| PM_2.5_ | Short-Term Exposure to PM_2.5_ Chemical Components... - Zhuang et al., 2024 | OR | 1.607 | (1.321, 1.956) |
| PM_2.5_ | The mitigation effects of residential green space and low air pollution... - Wu et al., 2025 | HR | 2.44 (combined effect) | (2.21, 2.71) |
| PM_2.5_ | Association Between Ambient Fine Particulate Matter and Physical Functioning... - Wang et al., 2022 | β, OR | Grip strength: -220g /60kg | (127, 312)g |
| PM_2.5_ | Correlation between air pollution and cognitive impairment... - Liu et al., 2023 | OR | Not the primary exposure (SO_2_, AQI were) | See SO_2_/AQI rows |
| PM_2.5_ | Cognitive impairment associated with individual and joint exposure... - Deng et al., 2024 | β | Reported for components | See component rows |
| PM_2.5_ | Impact of exposure to ambient air pollution on health related quality of life... - Xiao et al., 2025 | Not provided | Not provided | Not provided |
| PM_2.5_ | The impact of living environmental factors on cognitive function... - Luo et al., 2024 | HR | See tables | See tables |
| PM_2.5_ | Physical activity modifies the association between ambient air pollution... - Chen et al., 2025 | aHR | Multiple values | Multiple values |
| PM_2.5_ | Environmental determinants of depression risk in China... - Xie et al., 2025 | OR, HR | Multiple values | Multiple values |
| PM_2.5_ | Outdoor Air Pollution Exposure and Inter-relation... - Petkus et al., 2021 | β | β = -0.144 | (-0.261, -0.028) |
| PM_2.5_ | Associations of neighbourhood environmental attributes and socio-economic status... - Barnett et al., 2023 | Regression Coef. | -0.218 (on PCS) | (-0.393, -0.044) |
| PM_2.5_ | Long-term exposure to PM_2.5_ air pollution and mental health... - Lyons et al., 2024 | β, OR | CES-D: β=0.0312 | (0.0093, 0.0531) |
| PM_2.5_ | Air pollution and mental health... - Chen et al., 2023 | β | -0.243 (per μg/m³) | SE: 0.059 |
| PM_2.5_ | Disentangling impacts of multiple pollutants on acute cardiovascular events... - Humphrey et al., 2024 | Excess Risk % | Not the strongest pollutant (NO2 was) | See NO2 row |
| PM_2.5_ | Cohort profile: the Environmental-Pollution-Induced Neurological Effects... - Jang et al., 2021 | Not reported | Not reported | Not reported |
| PM_2.5_ | Long-term exposure to air pollution and risk of dementia... - Andersen et al., 2025 | HR | 1.14 | (1.12, 1.16) |
| PM_2.5_ | Railways to better minds... - Zong et al., 2025 | Indirect Effect | 0.038 | [0.026, 0.050] |
| PM_2.5_ | Population Health Effects of Air Pollution... - Shen et al., 2021 | β | -0.1512 (AQI's effect) | Not provided |
| PM_2.5_ | Assessing physical and mental health impacts of solid cooking fuel emissions - Kumar et al., 2025 | Cohen's d (range) | Wide range | Wide range |
| PM_2.5_ | Longitudinal mental health associations of relocation... - Sui et al., 2025 | β | -3.869 | [-7.583, -0.155] |
| PM_2.5_ | Exposures to ambient air pollutants increase prevalence of sleep disorder... - Zhou et al., 2023 | OR | 1.089 | (1.003, 1.182) |
| PM_2.5_ | Exposure and perception of PM_2.5_ pollution on the mental stress of pregnant women - Li et al., 2021 | OR | 1.1376 (per unit ADD) | (1.0826, 1.1953) |
| Note: Studies examining PM_2.5_ as a general metric without specifying components. | | | | |

Appendix table 8. Literature list of PM_2.5_ components studies **in older adults**

| **Exposure** | **Citation** | **E.M.** | **Point Estimate** | **95% CI** |
| --- | --- | --- | --- | --- |
| PM_2.5_ (BC, OM, SO_4_²^-^) | Long-term effects of fine particulate matter components on depression... - Liu et al., 2024 | HR | BC: 1.54;  OM: 1.24;  SO_4_²^-^: 1.25 | BC: (1.44, 1.64);  OM: (1.16, 1.34);  SO_4_²^-^: (1.16, 1.35) |
| PM_2.5_(OM, SO_4_²^-^, NH^4+^, etc.) | Short-Term Exposure to PM_2.5_ Chemical Components... - Zhuang et al., 2024 | OR | OM: 1.417;  SO_4_²^-^: 1.418;  NH^4+^: 1.025 | OM: (1.245, 1.612);  SO_4_²^-^: (1.247, 1.613);  NH^4+^: (1.009, 1.140) |
| PM_2.5_ (BC, OM, Sulfate, Nitrate, Ammonium) | Effects of short- and long-term exposures to multiple air pollutants... - Tong et al., 2024 | β, OR | BC (2-year): β=0.173, OR=1.126 | β: (0.007, 0.339);  OR: (1.125, 1.127) |
| PM_2.5_ (Nitrate, Ammonium, Sulfate, OC, BC) | Cognitive impairment associated with individual and joint exposure... - Deng et al., 2024 | β | Range: -1.477 (Nitrate) to -0.822 (BC) | See原文表1及图1 |
| BC | Long-term exposure to air pollution and risk of dementia... - Andersen et al., 2025 | HR | 1.23 | (1.20, 1.26) |
| Note: Studies examining specific chemical constituents of PM_2.5_ (e.g., Black Carbon, Organic Matter, Sulfate). | | | | |

Appendix table 9. Literature list of NO_2_ studies **in older adults**

| **Exposure** | **Citation** | **E.M.** | **Point Estimate** | **95% CI** |
| --- | --- | --- | --- | --- |
| NO_2_ | Associations Between Symptoms of Depression and Air Pollutant Exposure... - Wang et al., 2022 | OR | Part of NO_x_ analysis | See NO_x_ row |
| NO_2_ | Physical Activity-Induced Modification of the Association... - Park et al., 2024 | OR | 1.06 (per 1 ppb) | (1.04, 1.08) |
| NO_2_ | Association of Long-term Exposure to Air Pollution With Late-Life Depression... - Qiu et al., 2023 | HR | 1.006 | (1.003, 1.009) |
| NO_2_ | The increased risk of exposure to fine particulate matter for depression incidence... - Chang et al., 2025 | HR | Included in multi-pollutant model | Not primary focus |
| NO_2_ | Long-term effects of common air pollutants and depression... - Xiao et al., 2025 | HR | 1.03 | (0.98, 1.08) |
| NO_2_ | Activity-related dyspnea in older adults... - Verschoor et al., 2022 | OR | Part of multi-pollutant analysis | See table |
| NO_2_ | Effects of short- and long-term exposures to multiple air pollutants... - Tong et al., 2024 | β, OR | Included in analysis | Not primary focus |
| NO_2_ | Physical activity modifies the association between ambient air pollution... - Chen et al., 2025 | aHR | Multiple values | Multiple values |
| NO_2_ | Outdoor Air Pollution Exposure and Inter-relation... - Petkus et al., 2021 | β | β = -0.157 | (-0.291, -0.022) |
| NO_2_ | Associations of neighbourhood environmental attributes and socio-economic status... - Barnett et al., 2023 | Regression Coef. | Included in analysis | Not primary focus |
| NO_2_ | Disentangling impacts of multiple pollutants on acute cardiovascular events... - Humphrey et al., 2024 | Excess Risk % | 1.51% (per 10-unit) | (1.22%, 1.80%) |
| NO_2_ | Cohort profile: the Environmental-Pollution-Induced Neurological Effects... - Jang et al., 2021 | Not reported | Not reported | Not reported |
| NO_2_ | Long-term exposure to air pollution and risk of dementia... - Andersen et al., 2025 | HR | 1.25 | (1.22, 1.28) |
| NO_2_ | Assessing physical and mental health impacts of solid cooking fuel emissions - Kumar et al., 2025 | Cohen's d (range) | Wide range | Wide range |
| NO_2_ | Exposures to ambient air pollutants increase prevalence of sleep disorder... - Zhou et al., 2023 | OR | Included in analysis | Not primary focus |

Appendix table 10. Literature list of O_3_ studies **in older adults**

| **Exposure** | **Citation** | **E.M.** | **Point Estimate** | **95% CI** |
| --- | --- | --- | --- | --- |
| O_3_ | Associations Between Symptoms of Depression and Air Pollutant Exposure... - Wang et al., 2022 | OR | Included in multi-pollutant analysis | See table |
| O_3_ | Association of ambient ozone exposure with anxiety and depression... - Shi et al., 2022 | OR | Depression: 1.17 | (1.08, 1.27) |
| O_3_ | Association of Long-term Exposure to Air Pollution With Late-Life Depression... - Qiu et al., 2023 | HR | 1.021 | (1.016, 1.026) |
| O_3_ | The increased risk of exposure to fine particulate matter for depression incidence... - Chang et al., 2025 | HR | Included in multi-pollutant model | Not primary focus |
| O_3_ | Long-term effects of common air pollutants and depression... - Xiao et al., 2025 | HR | 0.59 | (0.57, 0.61) |
| O_3_ | Activity-related dyspnea in older adults... - Verschoor et al., 2022 | OR | Part of multi-pollutant analysis | See table |
| O_3_ | Effects of short- and long-term exposures to multiple air pollutants... - Tong et al., 2024 | β, OR | Included in analysis | Not primary focus |
| O_3_ | Long-term ambient ozone, omega-3 fatty acid... - Jin et al., 2024 | HR | Depression: 1.231 | (1.163, 1.303) |
| O_3_ | Impact of exposure to ambient air pollution on health related quality of life... - Xiao et al., 2025 | Not provided | Not provided | Not provided |
| O_3_ | Physical activity modifies the association between ambient air pollution... - Chen et al., 2025 | aHR | Multiple values | Multiple values |
| O_3_ | Disentangling impacts of multiple pollutants on acute cardiovascular events... - Humphrey et al., 2024 | Excess Risk % | Included in analysis | Not primary focus |
| O_3_ | The association between ozone and fine particles and mental health-related ED visits... - Nguyen et al., 2021 | % Change | 1.87% (7-day lag) | (0.62%, 3.15%) |
| O_3_ | 124. Exposures to ambient air pollutants increase prevalence of sleep disorder... - Zhou et al., 2023 | OR | Included in analysis | Not primary focus |

Appendix table 11. Literature list of indoor solid fuel studies **in older adults**

| **Exposure** | **Citation** | **E.M.** | **Point Estimate** | **95% CI** |
| --- | --- | --- | --- | --- |
| Solid fuel | The mediating effect of sleep quality on solid cooking fuel use... - Luo et al., 2024 | OR | 1.38 | (1.12, 1.70) |
| Solid fuel | The impact of new energy demonstration city policy... - Wan et al., 2025 | β | Included in policy analysis | Not primary focus |
| Solid fuel | Effects of long-term household air pollution exposure from solid fuel use... - Li et al., 2021 | HR | Heating: 1.27;  Cooking: 1.26 | Heating: (1.14, 1.42); Cooking: (1.13, 1.40) |
| Solid fuel | Associations of Indoor Ventilation Frequency with Depression and Anxiety... - Du et al., 2024 | OR | Included as a covariate/stratifier | Not primary focus |
| Solid fuel | Industrialization, indoor and ambient air quality, and elderly mental health - Ao et al., 2021 | β, OR | β=0.3363  (CES-D score) | SE reported |
| Solid fuel | Mediating Factors Explaining the Associations between Solid Fuel Use... - Yu et al., 2022 | β | -0.045 (depression as mediator) | SE: 0.007 |
| Solid fuel | The impact of living environmental factors on cognitive function... - Luo et al., 2024 | HR | See tables | See tables |
| Solid fuel | Environmental determinants of depression risk in China... - Xie et al., 2025 | OR, HR | Multiple values | Multiple values |
| Solid fuel | The effect of clean cooking on the social participation... - Li et al., 2024 | HR | 1.31  (clean vs. solid fuel) | (1.19, 1.44) |
| Solid fuel | Ecological Sustainability and Households’ Wellbeing... - Zhang et al., 2022 | ATT | -3.659 (score reduction) | Not provided |
| Solid fuel | Solid fuels use for cooking and sleep health... - Yu et al., 2021 | OR | 2.11  (sleep & depression co-occurrence) | (1.74, 2.55) |
| Solid fuel | Does clean cooking energy improve mental health?... - Liu et al., 2022 | DID Coef. | -1.522 (CES-D index) | Not reported |
| Solid fuel | Assessing physical and mental health impacts of solid cooking fuel emissions - Kumar et al., 2025 | Cohen's d (range) | Wide range | Wide range |

Appendix table 12. Literature list of green space studies **in older adults**

| **Exposure** | **Citation** | **E.M.** | **Point Estimate** | **95% CI** |
| --- | --- | --- | --- | --- |
| Green space | Impact of green infrastructure in smart older adult care communities... - Wang et al., 2025 | Regression Coef. | -0.14 (example) | Not provided |
| Green space | Association between air pollution (PM10, PM_2.5_), greenness and depression... - Park et al., 2024 | OR | Effect modifier  (NDVI Q4 vs Q1) | Reported in interaction |
| Green space | “Your soul will rest in the fresh air”... - Hawkins et al., 2025 | N/A (Qualitative) | N/A | N/A |
| Green space | China’s Healthy City Pilot Policy Improves Physical and Mental Health... - Zhang et al., 2025 | Beta | Part of policy pathway | Not isolated |
| Green space | The mitigation effects of residential green space and low air pollution... - Wu et al., 2025 | HR | Combined effect with low PM_2.5_: 2.44 | (2.21, 2.71) |
| Green space (Park access) | Associations between neighborhood park access and longitudinal change... - Besser et al., 2021 | OR | 1.04 (per 0.10 increase in park proportion) | (1.00, 1.08) |
| Green space | Associations of neighbourhood environmental attributes and socio-economic status... - Barnett et al., 2023 | Regression Coef. | Included in analysis | Not primary focus |
| Green space | Longitudinal mental health associations of relocation... - Sui et al., 2025 | β | Included in "supportive neighborhood" metric | Not isolated |
| Note: Studies examining green space exposure (e.g., NDVI, park access, green infrastructure). | | | | |

Appendix table 13. Literature list of other pollutants studies **in older adults**

| **Exposure** | **Citation** | **E.M.** | **Point Estimate** | **95% CI** |
| --- | --- | --- | --- | --- |
| PM_10_ | Ambient air pollution and the health-related quality of life... - Tan et al., 2023 | β | See dimension-specific analysis | Not provided |
| PM_10_ | Associations Between Symptoms of Depression and Air Pollutant Exposure... - Wang et al., 2022 | OR | Included in multi-pollutant analysis | See table |
| PM_10_ | Association between air pollution (PM_10_, PM_2.5_), greenness and depression... - Park et al., 2024 | OR | PM_10_ (NDVI Q1): 1.88 | (1.58, 2.25) |
| PM_10_ | The impact of healthy community environment and sleep quality... - Wei et al., 2025 | β | Included in "health environment" | Not isolated |
| PM_10_ | Fine particulate matter, vitamin D, physical activity, and major depressive disorder... - Wu et al., 2022 | OR | Included in analysis | Not primary focus |
| PM_10_ | Physical Activity-Induced Modification of the Association... - Park et al., 2024 | OR | Included in analysis | Not primary focus |
| PM_10_ | Air pollution, social engagement, and depression in older adults... - Wu et al., 2023 | HR | 1.07 (per 1 μg/m³) | (0.98, 1.18) |
| PM_10_ | Ambient air pollution and the health-related quality of life... - Tan et al., 2023 | β | Included in analysis | Not primary focus |
| PM_10_ | The increased risk of exposure to fine particulate matter for depression incidence... - Chang et al., 2025 | HR | Included in multi-pollutant model | Not primary focus |
| PM_10_ | Long-term effects of common air pollutants and depression... - Xiao et al., 2025 | HR | 1.31 | (1.24, 1.38) |
| PM_10_ | Association between ambient particulate matters and anhedonia... - Xie et al., 2024 | β | RSAS (PM_10_): 1.220 | (0.439, 2.00) |
| PM_10_ | Correlation between air pollution and cognitive impairment... - Liu et al., 2023 | OR | Included in multi-pollutant analysis | Not primary focus |
| PM_10_ | Impact of exposure to ambient air pollution on health related quality of life... - Xiao et al., 2025 | Not provided | Not provided | Not provided |
| PM_10_ | Physical activity modifies the association between ambient air pollution... - Chen et al., 2025 | aHR | Multiple values | Multiple values |
| PM_10_ | Cohort profile: the Environmental-Pollution-Induced Neurological Effects... - Jang et al., 2021 | Not reported | Not reported | Not reported |
| PM_10_ | Short-term attributable risk and economic burden... - Fu et al., 2025 | OR | Included in analysis | Not primary focus |
| PM_10_ | The association between ozone and fine particles and mental health-related ED visits... - Nguyen et al., 2021 | % Change | Included in analysis | Not primary focus |
| PM_10_ | Assessing physical and mental health impacts of solid cooking fuel emissions - Kumar et al., 2025 | Cohen's d (range) | Wide range | Wide range |
| PM_10_ | Exposures to ambient air pollutants increase prevalence of sleep disorder... - Zhou et al., 2023 | OR | 1.147 | (1.062, 1.240) |
| SO_2_ | Ambient air pollution and the health-related quality of life... - Tan et al., 2023 | β | See dimension-specific analysis | Not provided |
| SO_2_ | Associations Between Symptoms of Depression and Air Pollutant Exposure... - Wang et al., 2022 | OR | Included in multi-pollutant analysis | See table |
| SO_2_ | Ambient air pollution and the health-related quality of life... - Tan et al., 2023 | β | Included in analysis | Not primary focus |
| SO_2_ | The impact of environmental pollution on the physical health... - Fan et al., 2022 | Marginal Effect | Included in analysis | Not provided |
| SO_2_ | The increased risk of exposure to fine particulate matter for depression incidence... - Chang et al., 2025 | HR | Included in multi-pollutant model | Not primary focus |
| SO_2_ | Long-term effects of common air pollutants and depression... - Xiao et al., 2025 | HR | 1.45 | (1.38, 1.52) |
| SO_2_ | Activity-related dyspnea in older adults... - Verschoor et al., 2022 | OR | Part of multi-pollutant analysis | See table |
| SO_2_ | Correlation between air pollution and cognitive impairment... - Liu et al., 2023 | OR | Primary pollutant for cognition | (1.20899, 1.77116 for AQI) |
| SO_2_ | Impact of exposure to ambient air pollution on health related quality of life... - Xiao et al., 2025 | Not provided | Not provided | Not provided |
| SO_2_ | Long-term exposure to low-concentration sulfur dioxide and mental disorders... - Yuan et al., 2025 | HR | Depression: HR 1.11 (continuous) | (1.07, 1.16) |
| SO_2_ | Disentangling impacts of multiple pollutants on acute cardiovascular events... - Humphrey et al., 2024 | Excess Risk % | Included in analysis | Not primary focus |
| SO_2_ | Short-term attributable risk and economic burden... - Fu et al., 2025 | OR | Included in analysis | Not primary focus |
| SO_2_ | Assessing physical and mental health impacts of solid cooking fuel emissions - Kumar et al., 2025 | Cohen's d (range) | Wide range | Wide range |
| SO_2_ | Exposures to ambient air pollutants increase prevalence of sleep disorder... - Zhou et al., 2023 | OR | 1.158 | (1.065, 1.260) |
| CO | Associations Between Symptoms of Depression and Air Pollutant Exposure... - Wang et al., 2022 | OR | CO (7-day): 1.232 | (1.116, 1.361) |
| CO | The increased risk of exposure to fine particulate matter for depression incidence... - Chang et al., 2025 | HR | Included in multi-pollutant model | Not primary focus |
| CO | Long-term effects of common air pollutants and depression... - Xiao et al., 2025 | HR | 1.2 | (1.15, 1.25) |
| CO | Short-term attributable risk and economic burden... - Fu et al., 2025 | OR | Included in analysis | Not primary focus |
| CO | Assessing physical and mental health impacts of solid cooking fuel emissions - Kumar et al., 2025 | Cohen's d (range) | Wide range | Wide range |
| NO_x_ | Associations Between Symptoms of Depression and Air Pollutant Exposure... - Wang et al., 2022 | OR | NO_x_ (7-day): 1.312 | (1.158, 1.488) |
| NO_x_ | Air pollution, social engagement, and depression in older adults... - Wu et al., 2023 | HR | 1.26 (per 10 μg/m³) | (1.01, 1.58) |
| Noise | The impact of healthy community environment and sleep quality... - Wei et al., 2025 | β | Included in "health environment" | Not isolated |
| Noise | The increased risk of exposure to fine particulate matter for depression incidence... - Chang et al., 2025 | HR | Included in multi-pollutant model | Not primary focus |
| Noise | Feasibility of Deploying Home-Based Digital Technology... - Au-Yeung et al., 2024 | Spearman's ρ | Trends reported with anxiety | Not provided |
| Noise | Nonlinear effect of urban noise pollution on depression... - Jin et al., 2024 | β | Range: 0.1201 to 0.5709 | See文中表1 |
| Noise | Community Environment Perception on Depression... - Zhang et al., 2021 | β | Included in "community environment perception" | Not isolated |
| Drinking water quality | Associations among drinking water quality, dyslipidemia, and cognitive function... - Pan et al., 2022 | β | Global cognition: 0.58 | Not provided |
| Drinking water quality | Associations among drinking water quality, dyslipidemia, and cognitive function... - Pan et al., 2022 | β | Global cognition: 0.58 | Not provided |
| Composite index (AQI/API) | The impact of new energy demonstration city policy... - Wan et al., 2025 | β | Part of policy pathway | Not isolated |
| Composite index (AQI/API) | Correlation between air pollution and cognitive impairment... - Liu et al., 2023 | OR | 1.4633 (per unit) | (1.20899, 1.77116) |
| Composite index (AQI/API) | Population Health Effects of Air Pollution... - Shen et al., 2021 | β | -0.1512 (on mental health) | Not provided |
| Wildfire smoke (PM) | What can we do when the smoke rolls in?... - Humphreys et al., 2022 | N/A (Qualitative) | N/A | N/A |
| Allergens | Rediscovering Allergic Rhinitis... - Giuliano et al., 2022 | Median, P-value | Anxiety/Depression dimension median=2 (mHealth users) | N/A |
| Indoor ventilation frequency | Association between window ventilation frequency and depressive symptoms... - Luo et al., 2025 | OR | 0.67 | (0.51, 0.88) |
| Indoor ventilation frequency | Associations of Indoor Ventilation Frequency with Depression and Anxiety... - Du et al., 2024 | OR | Depression (high freq.): 0.49 | (0.43, 0.57) |
| Coal power plant emissions | Effect of residential proximity to the lignite-fired power plant... - Mata et al., 2022 | Regression Coef. | 1.003 (<10km vs >15km, crude) | (-0.313, 2.319) |
| Wastewater discharge | The impact of environmental pollution on the physical health... - Fan et al., 2022 | Marginal Effect | Included in analysis | Not provided |
| Mn | Cohort profile: the Environmental-Pollution-Induced Neurological Effects... - Jang et al., 2021 | Not reported | Not reported | Not reported |
| TVOCs, CO_2_, Temp, Humidity, Light | Feasibility of Deploying Home-Based Digital Technology... - Au-Yeung et al., 2024 | Spearman's ρ | Trends reported | Not provided |
| Note: Studies examining other specific pollutants not covered above (e.g., PM_10_, SO_2_, CO, noise, water quality, composite indices). | | | | |

Appendix table 14. Author, publication year, exposure, outcome and key findings of all 80 included studies in the review

| **ID** | **Author (Year)** | **Country/Region** | **Study Design** | **Sample (Age)** | **Main Exposure** | **Main Outcome** | **Key Finding** | **Tags** |
| --- | --- | --- | --- | --- | --- | --- | --- | --- |
| 1 | Yao et al., 2022 | China | Cross-sectional | 15,105; ≥45 y | Long-term ambient PM_2.5_ (30d to 2y moving averages) | Depressive symptoms (CESD-10 score ≥10) | 180d PM_2.5_ had the strongest positive association (OR=1.07, 95% CI:1.03-1.12 per 10µg/m^3^); stronger in non-drinkers, low physical activity groups, western residents | Short-Term Exposure; High-Pollution Area; PM_2.5_ |
| 2 | Wang et al., 2025 | China | Longitudinal panel | 2,400; ≥60 y | GI (coverage, uniformity, connectivity, accessibility, smart older adult care communities) | Physical health (chronic/acute disease index); Mental health (depression, anxiety, subjective wellbeing); Social interaction frequency; COVID-19 prevalence | Increased GI coverage linked to reduced chronic/acute diseases (*β*=-0.34, *p*<0.01), lower depression (*β*=-0.14), lower anxiety (*β*=-0.12), higher subjective wellbeing (*β*=0.45), more social interaction (*β*=0.29, *p*<0.01) and lower COVID-19 prevalence; stronger in males, 70-79 years | High-Pollution Area; Green Space |
| 3 | Luo et al., 2024 | China (Shandong, Rural) | Cross-sectional | 3,240; ≥60 y | Household solid cooking fuels vs clean fuels (coal, wood/straw) | Psychological distress (K10 score ≥21) | Solid cooking fuel use associated with higher psychological distress (OR=1.38, 95% CI:1.12-1.70); PSQI mediated 16.18% (indirect effect *β*=0.06, *p*=0.011) | High-Pollution Area; Rural Environment; Indoor Solid Fuel |
| 4 | Tan et al., 2023 | China (Shandong) | Cross-sectional | 5,717; ≥65 y | Long-term ambient PM_2.5_, PM_10_, SO_2_ (5-year average) | HRQoL (EQ-5D-3L utility score + 5 dimensions: mobility, self-care, usual activities, pain/discomfort, anxiety/depression) | Per 1μg/m^3^ increase: PM_2.5_ reduced EQ-5D-3L score by 0.002 ( *p*<0.01, pain/discomfort/anxiety/depression), PM_10_ reduced score by 0.001 (p<0.01, anxiety/depression), SO_2_ reduced score by 0.002 (p<0.01, pain/discomfort); stronger in higher SES groups | Long-Term Exposure; High-Pollution Area; Urban Environment; PM_2.5_; PM_10_; SO_2_ |
| 5 | Hu et al., 2023 | China (Nationally Representative) | Cross-sectional | 4,190; ≥60 y | Multidimensional health indicators (ADLs, IADLs, chronic diseases, depressive symptoms, cognitive function, social participation) | Latent classes of multidimensional health status (LCA) | Three latent health classes identified: Relatively Healthy (66.97%), Highly Depressed/Health Risk (28.38%), Functional Impairment (4.65%); illiteracy linked to High Depression class (OR=2.216, 95% CI:1.753-2.800); key determinants: no spouse, inactivity, low savings, rural residence, low air quality satisfaction | Long-Term Exposure; High-Pollution Area |
| 6 | Wang et al., 2022 | China (Taiwan) | Repeated-measures cohort | 1,956; ≥65 y | Short & long-term ambient PM_10_, O^3^, SO_2_, CO, NO, NO_2_, NO_x_ (7d to 1y moving averages) | Moderate-to-severe depressive symptoms (CESD-10 score ≥10) | CO/NO_x_ associated with higher depressive symptoms across all windows (1-year CO: OR=1.180, 95% CI:1.102-1.265; 1-year NO_x_: OR=1.219, 95% CI:1.130-1.314); only NO_x_ significant in two-pollutant models; cognitive decline amplified PM_10_/SO_2_ risks | Short-Term Exposure; High-Pollution Area; NO_2_; O^3^; PM_10_; SO_2_; CO; NO_x_ |
| 7 | Park et al., 2024 | South Korea | Longitudinal panel | 7,657; ≥45 y | Long-term ambient PM_2.5_, PM_10_; residential greenness (NDVI) | Depressive symptoms (CESD-10 score ≥20) | Higher NDVI attenuated air pollution-depression association; 10μg/m^3^ PM_10_: OR=1.88 (95% CI:1.58-2.25) (Q1) vs OR=1.29 (95% CI:1.06-1.58) (Q4); 10μg/m^3^ PM_2.5_: OR=3.75 (95% CI:2.75-5.10) (Q1) vs OR=1.78 (95% CI:1.30-2.44) (Q4) | Long-Term Exposure; Urban Environment; PM_2.5_; Green Space; PM_10_ |
| 8 | Wei et al., 2025 | China | Cross-sectional | 696; ≥65 y | Self-reported healthy community environment (environment, services, neighborhood) | Physical health (subjective and objective measures) | Sleep quality mediated healthy environment-objective physical health (95% CI:0.007-0.043) and healthy neighborhood-subjective physical health (95% CI:0.005-0.064) | Long-Term Exposure; Urban Environment; PM_2.5_; PM_10_; Noise |
| 9 | Wan et al., 2025 | China | Quasi-experimental (DID) | 9,381; ≥45 y | NEDC designation (2014) | Self-rated health (SRH); Depressive symptoms (CESD-10 scale) | NEDC policy improved SRH (*β*=0.0462, *p*<0.01) and reduced depressive symptoms (*β*=-0.1580, *p*<0.1); 2SLS confirmed causal effects (SRH *β*=1.2105, *p*<0.01; CESD-10 *β*=-7.7541, *p*<0.01) | Long-Term Exposure; High-Pollution Area; Urban Environment; Indoor Solid Fuel; Composite Index (AQI/API) |
| 10 | Wu et al., 2022 | UK | Cross-sectional | 39,168; ≥60 years | Long-term ambient PM_2.5_ (2010 annual estimate) | Lifetime major depressive disorder (mild/moderate/severe) | PM_2.5_ highest vs lowest quartile: OR=1.096, 95% CI:1.023-1.175; per 10μg/m^3^ increase: OR=1.399, 95% CI:1.070-1.830; vitamin D/physical activity attenuated PM_2.5_-depression association | Long-Term Exposure; Low-Pollution Area; PM_2.5_; PM_10_ |
| 11 | Sun et al., 2021 | China | Cross-sectional | 1,924; ≥45 y | URRBMI coverage | Income-related inequality in depressive symptoms (occurrence and score) | URRBMI reduced pro-rich depression occurrence inequality (18.88%) and score inequality (3.55%); URRBMI linked to lower depression (OR=0.693, *p*<0.01) | High-Pollution Area; PM_2.5_ |
| 12 | Pan et al., 2022 | China | Cross-sectional | 4,951; ≥60 y | Drinking water quality (Blue City Index: Excellent/Good vs Moderate/Poor) | Cognitive function (mental status, episodic memory, global cognition) | High drinking water quality linked to better mental status (*β*=0.34, *p*<0.001), episodic memory (*β*=0.24, *p*<0.05), global cognition (*β*=0.58, *p*<0.01); self-reported dyslipidemia acted as a suppressor | Long-Term Exposure; High-Pollution Area; Drinking Water Quality |
| 13 | Park et al., 2022 | South Korea | Cross-sectional | 1,454; ≥50 y | Long-term ambient NO_2_, PM10, PM_2.5_ (5-year average) | Depression (GDS-SF score ≥8) | Per 1ppb NO_2_ increase: OR=1.06, 95% CI:1.04-1.08 for depression; stronger in ≥65 years high physical activity group (OR=1.21, 95% CI:1.09-1.33 vs inactive OR=1.04, 95% CI:1.01-1.07, p-interaction=0.004) | Long-Term Exposure; Urban Environment; PM_2.5_; NO_2_; PM_10_ |
| 14 | Wu et al., 2023 | Sweden | Population-based longitudinal cohort | 2,812; ≥60 y | Long-term ambient PM_2.5_, PM_10_, NO_x_ (3-year moving average) | Incident depression (DSM-IV-TR criteria) | Per 1µg/m^3^ PM_2.5_ increase: HR=1.53, 95% CI:1.22-1.93 for incident depression; association attenuated in socially active participants (HR=1.04, 95% CI:0.70-1.55) | Long-Term Exposure; Low-Pollution Area; PM_2.5_; PM_10_; NO_x_ |
| 15 | Luo et al., 2024 | China | Cross-sectional | 7,887; ≥65 y (mean 82.6 y) | Indoor window ventilation frequency (low/medium/high, annual score) | Depressive symptoms (CESD-10 score >10) | High ventilation frequency linked to 33% lower depressive symptoms (OR=0.67, 95% CI:0.51-0.88) vs low frequency | High-Pollution Area; Indoor Ventilation Frequency |
| 16 | Tan et al., 2023 | China (Shandong) | Cross-sectional | 5,717; ≥65 y (mean 71.85 y) | Long-term ambient PM_2.5_, PM_10_, SO_2_ (5-year average) | HRQoL (EQ-5D-3L utility score + dimensions) | Per 1µg/m^3^ increase: PM_2.5_ reduced EQ-5D-3L score by 0.002 (p<0.05), PM_10_ by 0.001 (p<0.05), SO_2_ by 0.002 (p<0.05); air pollution linked to more pain/discomfort and anxiety/depression | Long-Term Exposure; High-Pollution Area; Urban Environment; PM_2.5_; PM_10_; SO_2_ |
| 17 | Li et al., 2021 | China | Longitudinal cohort (4 waves:2011-2018) | 7,005; ≥45 y | Household solid fuel use (heating/cooking, duration/fuel type, crop residue/wood/coal) | Depressive symptoms (CESD-10 score >12) | Solid fuel use linked to higher depression (heating: HR=1.27, 95% CI:1.14-1.42; cooking: HR=1.26, 95% CI:1.13-1.40); ≥7 years exposure increased risk; crop residue/wood had stronger effects | Long-Term Exposure; High-Pollution Area; Indoor Solid Fuel |
| 18 | Shi et al., 2022 | China | Multi-center repeated measurement | 3,445; mean 63.7 y (≥40 y) | Short-term ambient O^3^ (8h maximum, 2w-3m moving averages) | Anxiety (GAD-7 score ≥5); Depression (PHQ-9 score ≥5) | Per 10μg/m^3^ 3-month O^3^ increase: anxiety OR=1.25, 95% CI:1.15-1.37, depression OR=1.17, 95% CI:1.08-1.27; high temperature enhanced O^3^-anxiety association | Short-Term Exposure; High-Pollution Area; O^3^ |
| 19 | Shao et al., 2021 | China | Prospective cohort | 8,637; mean 57.8 y | Household solid fuel use (cooking/heating) | Incident depressive symptoms (CESD score ≥12) | Solid fuel for cooking+heating: HR=1.15, 95% CI:1.01-1.31; cooking only: HR=1.12, 95% CI:1.02-1.24; stronger in males (HR=1.24) and smokers (HR=1.32) | High-Pollution Area; Indoor Solid Fuel |
| 20 | Abaszadeh et al., 2024 | Iran | Parallel RCT | 36; ≥65 y (mean 65.78 y, SD=2.41) | PAAIR intervention (12w, 2×/week, 45min: physical activity + Amygdala/Insula Retraining) | Sleep quality (PSQI); Depressive symptoms (BDI); Working memory (N-back); Emotion regulation (DERS) | PAAIR improved sleep quality (t=7.63, *p*<0.001, d=0.98), reduced depressive symptoms (t=19.46, *p*<0.001, d=2.65), improved working memory (t=6.11, *p*<0.001, d=1.71) and emotion regulation (t=13.23, *p*<0.001, d=2.77); effects sustained at 8w follow-up | Intervention; Physical Activity; Sleep Quality; Mental Health |
| 21 | Luo et al., 2024 | China | Cross-sectional | 12,096; ≥65 y | Indoor ventilation frequency (low/intermediate/high, annual score) | Depression (CESD-10 ≥10); Anxiety (GAD-7 ≥10) | High vs low ventilation: depression OR=0.49, 95% CI:0.43-0.57, anxiety OR=0.63, 95% CI:0.43-0.91; intermediate vs low: depression OR=0.65, anxiety OR=0.55 | High-Pollution Area; Urban Environment; Indoor Solid Fuel; Indoor Ventilation Frequency |
| 22 | Qiu et al., 2023 | USA | Population-based longitudinal cohort | 8,907,422; ≥73.7 y (SD=4.8) | Long-term ambient PM_2.5_, NO_2_, O^3^ (5-year moving average) | Late-life depression (ICD-9/10) | Per 5-unit increase: PM_2.5_ increased late-life depression risk by 0.91% (HR=1.009, 95% CI:0.02%-1.81%), NO_2_ by 0.61% (HR=1.006, 95% CI:0.31%-0.92%), O^3^ by 2.13% (HR=1.021, 95% CI:1.63%-2.64%) | Long-Term Exposure; Low-Pollution Area; Urban Environment; PM_2.5_; NO_2_; O^3^ |
| 23 | Chen et al., 2024 | USA | Cross-sectional | 8,138; ≥20 y | Serum BFRs (PBB153, PBDEs etc.) | Depression (PHQ-9 score ≥10) | PBB153 Q4 vs Q1: OR=1.787, 95% CI:1.200-2.661 for depression; BFRs mixture per decile increase: OR=1.176, 95% CI:1.054-1.313; stronger in males (OR=1.429) | BFRs |
| 24 | Chang et al., 2025 | China (Taiwan) | Longitudinal cohort | 2,998; ≥55 y (mean ~68.4 y) | Long-term ambient PM_2.5_, PM_10_, CO, O^3^, NO_2_, SO_2_; road traffic noise (Lden) | Newly diagnosed depressive disorder (ICD-9-CM/ICD-10-CM) | Per IQR PM_2.5_ increase (8.53μg/m^3^): HR=1.175, 95% CI:1.012-1.363 for depressive disorder; association partially mediated by serum TNF-R1 (average mediated effect *β*=-0.961, 95% CI:-3.163 to -0.021) | Long-Term Exposure; Urban Environment; PM_2.5_; NO_2_; O^3^; PM_10_; SO_2_; CO; Noise |
| 25 | Ao et al., 2021 | China | Population-based longitudinal cohort | 28,962; ≥45 y (mean ~60.6 y) | Long-term ambient PM_2.5_ (satellite-based); household solid cooking fuel use | Mental health (CESD-10 score; depressive symptoms ≥10) | Per 1μg/m^3^ PM_2.5_ increase: CESD-10 score +0.0237 (p<0.05), depressive symptoms probability +0.24pp (p<0.01); solid fuel use: score +0.3363 (p<0.01), probability +2.29pp (p<0.05); stronger in less educated females | Long-Term Exposure; High-Pollution Area; Urban Environment; PM_2.5_; Indoor Solid Fuel |
| 26 | Yu et al., 2022 | China | Cross-sectional (SEM) | 7,831; ≥65 y | Household solid cooking fuel use (coal/charcoal/wood vs clean fuels) | Self-rated health (5-point Likert scale) | Solid fuel use linked to poorer SRH (total effect *β*=-0.107, *p*<0.001); mediated by sleep quality (*β*=-0.013), cognitive abilities (*β*=-0.003), depressive symptoms (*β*=-0.045, strongest mediator) | Long-Term Exposure; High-Pollution Area; Indoor Solid Fuel |
| 27 | Xiao et al., 2025 | China | Prospective cohort (CHARLS 2011–2020) | 8,131; ≥45 y (mean 62.04 y) | Long-term ambient PM_2.5_, PM_10_, O^3^, NO_2_, SO_2_, CO (1km resolution) | Incident depression (CESD-10 ≥10) | Per IQR increase: PM_2.5_ HR=1.42, 95% CI:1.35-1.50, PM_10_ HR=1.31, 95% CI:1.24-1.38, SO_2_ HR=1.45, 95% CI:1.38-1.52, CO HR=1.20, 95% CI:1.15-1.25 for incident depression; O^3^ inversely associated (HR=0.59, 95% CI:0.57-0.61) | Long-Term Exposure; High-Pollution Area; PM_2.5_; NO_2_; O^3^; PM_10_; SO_2_; CO |
| 28 | Hawkins et al., 2025 | USA (Southeastern Wisconsin) | Qualitative (Narrative Inquiry/Photovoice) | 23; ≥60 y (mean 76 y) | Place-specific factors (built environment, green space, social support, discrimination) | Self-reported health and wellbeing | Parks/green space/social support positively impacted mental health; outsiderness/discrimination negatively affected wellbeing; no quantitative effect sizes reported | Long-Term Exposure; Green Space |
| 29 | Dales et al., 2022 | Canada | Cross-sectional | 28,854; ≥45 y | Ambient O^3^, NO_2_, PM_2.5_, SO_2_; sociodemographic/comorbidity factors | Activity-related dyspnea (level 2: stairs/uphill; level3: flat surface) | Adjusted OR for level3 dyspnea: obesity 5.71, lung disease 3.91, depression 3.68; joint effects: depression+heart disease OR=18.31, depression+lung disease OR=12.78; O^3^/NO_2_ had small significant associations | Long-Term Exposure; Low-Pollution Area; NO_2_; O^3^; SO_2_ |
| 30 | Mata et al., 2022 | Thailand (Lampang Province) | Cross-sectional | 130; ≥60 y | Residential proximity to lignite-fired power plant (<10km/10–15km/>15km) | Depression (PHQ-9); Sleep quality (PSQI); Morning salivary cortisol | Living <10km vs >15km from power plant: lower cortisol (*β*=-0.320, 95% CI:-0.460 to -0.179, *p*<0.001), poorer PSQI (*β*=1.350, 95% CI:0.265-2.436, *p*=0.015); no significant depression association | Long-Term Exposure; High-Pollution Area; Coal Power Plant Emissions |
| 31 | Do et al., 2025 | USA | Case-crossover | 927,581; ≥65 y (83.9%>75 y) | Short-term ambient wildfire PM_2.5_ (daily, 10km×10km, lag 0–6d) | Cause-specific hospitalizations (circulatory/respiratory/anxiety/depression) | Null association for circulatory/respiratory/anxiety hospitalizations; same-day PM_2.5_ (10μg/m^3^): depression hospitalization RR=0.94, 95% CI:0.90-0.99; effect modified by urbanicity (rural positive; urban null) | Short-Term Exposure; PM_2.5_ |
| 32 | Liu et al., 2024 | China | Nationwide cohort (CHARLS 2011–2018) | 5,707; ≥45 y (median 57.0 y, IQR=13) | Long-term ambient PM_2.5_ components (BC, NH_4_^+^, NO^3-^, OM, SO_4_^2-^, 1-3y moving averages) | Incident depression (CESD-10 ≥10) | Per IQR 3-year average increase: BC HR=1.54, 95% CI:1.44-1.64, OM HR=1.24, 95% CI:1.16-1.34, SO_4_^2-^ HR=1.25, 95% CI:1.16-1.35 for incident depression; stronger in females, younger adults, urban residents | Long-Term Exposure; High-Pollution Area; PM_2.5_; PM_2.5_ Components |
| 33 | Grineski et al., 2025 | USA | Longitudinal cohort (MIDUS 2-3) | 5,000; 32–84 y (mean 55 y at M2) | Long-term ambient PM_2.5_ (5-year annual average) | Executive functioning (BTACT EF composite); Functional limitations | Per 10μg/m^3^ PM_2.5_ increase: lower executive functioning cross-sectionally (*β*=-0.11, *p*<0.05) and longitudinally (*β*=-0.13, *p*<0.05); cardiometabolic disease partially mediated (indirect *β*=-0.009, *p*=0.048) | Long-Term Exposure; PM_2.5_ |
| 34 | Pan et al., 2022 | China | Cross-sectional (path analysis) | 4,951; ≥60 y (mean 68.1 y) | Drinking water quality (Blue City Index: Excellent/Good vs Moderate/Poor) | Cognitive function (mental status, episodic memory, global cognition) | High drinking water quality linked to better mental status (*β*=0.34, *p*<0.001), episodic memory (*β*=0.24, *p*<0.05), global cognition (β=0.58, *p*<0.01); self-reported dyslipidemia acted as a suppressor | High-Pollution Area; Drinking Water Quality |
| 35 | Au-Yeung et al., 2024 | USA (Oregon) | Longitudinal observational feasibility | 9; ≥60 y (mean 76.3 y, SD=5.1) | Digital biomarkers; indoor environmental factors (light, noise, CO2, TVOC, PM_2.5_) | Neuropsychiatric symptoms (apathy, depression, anxiety) | Feasibility confirmed: 98.9% survey adherence, 89.5%-90.4% sensor data capture; sleep onset duration correlated with anxiety; noise linked to anxiety in 3/9 participants (*p*=0.005-0.02); no effect sizes reported | Short-Term Exposure; PM_2.5_; Noise; TVOCs; CO2 |
| 36 | Xie et al., 2023 | China (Anhui) | Cross-sectional | 538; 18–65 y (mean 41.2 y, SD=13.1) | Long-term ambient PM_2.5_, PM_10_ (1-24m exposure windows) | Anhedonia (social: RSAS; physical: RPAS) | Per IQR PM_2.5_ increase: 12m RSAS *β*=1.238, 95% CI:0.353-2.123, 18m RPAS *ββ*=1.888, 95% CI:0.699-3.078; PM_10_ had similar positive associations; stronger in females, <40 years, low income | Long-Term Exposure; High-Pollution Area; PM_2.5_; PM_10_ |
| 37 | Tong et al., 2024 | China | Nationwide longitudinal (CLDS 2016-2018) | 27,457; 15–64 y (mean43.0 y) | Short & long-term ambient PM_2.5_, PM_2.5_ components, O^3^ (1-3m/1-2y) | Depression (CESD score; prevalence ≥16) | Per 10μg/m^3^ 2-year average increase: BC OR=1.126, 95% CI:1.125-1.127, PM_2.5_ OR=1.005, 95% CI:1.004-1.006, O^3^ OR=1.007, 95% CI:1.003-1.010 for depression; stronger in workers without labor contracts/low income | Short-Term Exposure; High-Pollution Area; PM_2.5_; PM_2.5_ Components; NO_2_; O^3^ |
| 38 | Jin et al., 2024 | UK | Prospective cohort (UK Biobank) | 257,534; 37–73 y (mean56.6 y, SD=8.0) | Long-term ambient O^3^ (annual mean) | Incident depression/anxiety (ICD-10: F32-F33/F40-F41) | Per 10μg/m^3^ O^3^ increase: depression HR=1.231, 95% CI:1.163-1.303, anxiety HR=1.237, 95% CI:1.168-1.310; higher omega-3 intake attenuated O^3^-depression association (P-interaction=0.012) | Long-Term Exposure; Low-Pollution Area; O^3^ |
| 39 | Liao et al., 2025 | China (Taiwan) | Cross-sectional (Taiwan Biobank) | 25,593; ≥60 y (mean63.0 y, SD=3.5) | Long-term ambient PM_2.5_ (5-year average, 1km resolution) | Cognitive performance (MMSE score, 0-30) | Per 1μg/m^3^ PM_2.5_ increase: MMSE β=-0.0830, 95% CI:-0.0973 to -0.0688, *p*<0.0001; cognitive performance PRS linked to higher MMSE; schizophrenia/AD PRS linked to lower MMSE; no gene-environment interaction | Long-Term Exposure; High-Pollution Area; PM_2.5_ |
| 40 | Wang et al., 2025 | China (Beijing) | Cross-sectional community-scale | 806; ≥60 y (mean72.2 y, SD=6.9) | Urban landscape (BD/BH/GD etc.); environmental stress (PM_2.5_/PET) | Depression (GDS-15); Anxiety (GAI) | Environmental stress (PM_2.5_/PET) contributed more to depression (12.29%/11.90%) and anxiety (12.77%/11.92%) than urban landscape; thresholds: PM_2.5_>97–99μg/m^3^, PET>27.6–27.8°C increased mental health risk | Long-Term Exposure; High-Pollution Area; Urban Environment; PM_2.5_ |
| 41 | Zhang et al., 2025 | China | Quasi-natural experiment (PSM-DID, CHARLS 2011–2020) | 16,871; ≥45 y (mean60.66 y) | HCPP implementation (2016) | Physical health (SRH/medical expenses); Mental health (life satisfaction/depression) | HCPP improved SRH (*β*=0.374, *p*<0.001), reduced medical expenses (*β*=-0.449, *p*<0.001), increased life satisfaction (*β*=0.783, *p*<0.001), reduced depression (*β*=-0.957, *p*<0.001); mediated by more physical/social activity and lower PM_2.5_ | Long-Term Exposure; High-Pollution Area; Urban Environment; PM_2.5_; Green Space |
| 42 | BAdeagbo et al., 2025 | India | Cross-sectional (WHO-SAGE Wave2) | 4,214; ≥60 y (mean68.4 y, SD=7.0) | Asthma (physician-diagnosed/symptom-based) | Depression (ICD-10); Loneliness; Sleep difficulties | Physician-diagnosed asthma: depression AOR=2.10, 95% CI:1.41-3.14, sleep difficulties AOR=1.61, 95% CI:1.07-2.41; symptom-based asthma: depression AOR=2.17, loneliness AOR=1.73, sleep difficulties AOR=2.20; rural residence amplified asthma-sleep association | High-Pollution Area |
| 43 | Zhuang et al., 2024 | China (Huizhou/Shenzhen/Zhaoqing) | Time-stratified case-crossover | 247,281; 10.57%≥60 y | Short-term ambient PM_2.5_, PM_2.5_ components (BC/OM/SO_4_^2-^ etc., lag 0-21d) | Daily outpatient visits for depression (ICD-10: F32-F33) | Lag0-21 cumulative OR (50th percentile): PM_2.5_=1.607, 95% CI:1.321-1.956, OM=1.417, 95% CI:1.245-1.612 for depression outpatient visits; stronger in females and the elderly | Short-Term Exposure; High-Pollution Area; PM_2.5_; PM_2.5_ Components |
| 44 | Fenwick et al., 2023 | Singapore | Cross-sectional population-based | 2,503; ≥60 y (mean73.4 y, SD=8.4) | Hearing impairment (laterality/severity) | H-QoL (HHIE-S); Depressive symptoms (PHQ-9≥6); Cognitive impairment; Gait speed | Bilateral severe HI vs no HI: worse HHIE-S (*β*=16.78, 95% CI:13.25-20.31, *p*<0.001), slower gait speed (*β*=-0.11, 95% CI:-0.19 to -0.04, *p*=0.003); no significant depression/cognitive impairment association (Bonferroni corrected) | Urban Environment |
| 45 | Wu et al., 2025 | UK | Prospective cohort (UK Biobank) | 334,536; 37–73 y (mean56.3 y, SD=8.1) | SES; residential green space; long-term ambient PM_2.5_ | Incident depression (ICD-10: F32-F33) | Low SES linked to higher depression (HR=1.30, 95% CI:1.27-1.32); green space+low PM_2.5_ mediated 2.7% of the association; low SES in favorable environments had 14.6% lower risk than medium SES in unfavorable environments | Long-Term Exposure; Low-Pollution Area; PM_2.5_; Green Space |
| 46 | Giuliano et al., 2022 | Italy | Cross-sectional observational pilot | 94; 65–93 y (mHealth:71.6±4.7 y; non-mHealth:73.8±4.5 y) | Allergic rhinitis; MASK-air® mHealth app usage | HRQoL (EQ-5D-5L) | mHealth users had worse anxiety/depression scores (*p*=0.018); females had worse mobility and anxiety/depression scores (p<0.05); AR impacted daily activities in 57.4% of participants | Allergens |
| 47 | Wang et al., 2020 | China | Nationwide longitudinal cohort (CHARLS 2011-2015) | 10,823; ≥45 y (mean60.1 y, SD=9.0) | Long-term ambient PM_2.5_ (12-month moving average) | Physical functioning (grip strength/balance/chair stands/gait speed) | Per 10μg/m^3^ PM_2.5_ increase: grip strength -220g/60kg (95% CI:-312 to -127), balance OR=0.95, 95% CI:0.93-0.98; effects equivalent to 1.12 years (grip) and 0.98 years (balance) of aging | Long-Term Exposure; PM_2.5_ |
| 48 | Liu & Hu, 2023 | China (122 Cities) | Longitudinal panel (CHARLS 2015-2018) | 28,395; ≥60 y (mean~68.0 y in 2015) | Air pollution (AQI, SO_2_, NO_2_, PM_10_) | Cognitive impairment (CSI-D, binary) | Higher AQI linked to cognitive impairment (OR=1.4633, 95% CI:1.20899-1.77116); SO_2_ OR=1.3802, 95% CI:1.25779-1.51451; stronger in ≥70 years, females, lower education; social adaptability moderated the association | Long-Term Exposure; Overall PM_2.5_; PM_10_; SO_2_; Composite Index (AQI/API) |
| 49 | Deng et al., 2024 | China | Nationwide dynamic cohort (CHARLS 2011–2018) | 13,507; ≥45 y (mean56.6 y, SD=8.7) | Long-term ambient PM_2.5_, PM_2.5_ components (BC/OM/NO^3-^ etc.) | Cognitive function (global/episodic memory/mental status) | Per IQR component increase: global cognition *β*=-1.477 (NO^3-^), -1.331 (NH_4_^+^), -1.033 (SO_4_^2-^), -0.988 (OM), -0.822 (BC); joint exposure *β*=-1.353, 95% CI:-1.659 to -1.048; stronger in females, 45-64 years, high education | Long-Term Exposure; PM_2.5_; PM_2.5_ Components |
| 50 | Besser et al., 2025 | USA (6 Cities) | Longitudinal cohort (MESA) | 1,733; ≥50 y (mean67.3 y, SD=8.3) | Neighborhood park access (park space proportion/distance to park) | 6-year change in cognition (global/processing speed) | Per 10% park space increase (½-mile): OR=1.04, 95% CI:1.00-1.08 for maintained/improved global cognition; borderline association in African Americans (OR=1.07, 95% CI:1.00-1.14) | Long-Term Exposure; Low-Pollution Area; Green Space |
| 51 | Jin et al., 2025 | China (85 Prefecture-Level Cities) | Longitudinal (CHARLS 2011–2020) | Unspecified; ≥65 y | Urban noise pollution (annual ESPL, dB(A)) | Depression score (CESD-10, 0–30) | High vs low ESPL: depression score +2.28 (RD, *p*<0.001), depression risk +13.9% (RR=1.139); U-shaped dose-response: ESPL<53.67dB(A) beneficial; >55.68dB(A), score +0.85 per 1dB(A) | Long-Term Exposure; Urban Environment; Noise |
| 52 | Xiao et al., 2025 | China (Ningxia, Rural) | Cross-sectional | 4,349; ≥60 y (mean69.6 y, SD=7.2) | Long-term ambient PM_2.5_, PM_10_, SO_2_, O^3^ (3-year average) | HRQoL (EQ-5D-3L utility score + 5 dimensions) | Per 1μg/m^3^ SO_2_ increase: EQ-5D-3L score -0.010 (p<0.05); PM_2.5_/PM_10_/O^3^ had negative non-significant associations; nonlinear dose-response for SO_2_ with all 5 dimensions; PM_2.5_/PM_10_ with self-care/usual activities | Long-Term Exposure; Rural Environment; PM_2.5_; O^3^; PM_10_; SO_2_ |
| 53 | Luo et al., 2024 | China | Cross-sectional & longitudinal cohort (CHARLS 2011–2018) | Cross:4,401; ≥60y (mean67.2 y); Long:3,177 (MCI-free at baseline) | Living environmental factors (composite score: PM_2.5_, fuel type, water source etc.) | Cognitive function; Incident MCI (AACD criteria) | Low vs high risk environment: higher global cognition (*β*=1.25, 95% CI:0.85-1.65), lower MCI risk (HR=0.67, 95% CI:0.49-0.91); protective factors: clean fuels (HR=0.74), tap water (HR=0.84) | Long-Term Exposure; PM_2.5_; Indoor Solid Fuel |
| 54 | Chen et al., 2025 | China | Nationwide longitudinal cohort (CHARLS 2011–2020) | 11,875; ≥45 y (54.6% female) | Long-term ambient PM1, PM_2.5_, PM_10_, O^3^, NO_2_ (1-year lag) | Incident depression, ADL disability, their comorbidity | Per 10μg/m^3^ increase: NO_2_ aHR=1.43, 95% CI:1.32-1.54, PM1 aHR=1.32, PM_2.5_ aHR=1.15, O^3^ aHR=1.13, PM_10_ aHR=1.07 for depression-ADL comorbidity; physical activity attenuated pollutant associations | Long-Term Exposure; High-Pollution Area; PM_2.5_; NO_2_; O^3^; PM_10_ |
| 55 | Xie et al., 2025 | China | Cross-sectional & longitudinal cohort (CHARLS 2011–2018) | Cross:11,184; ≥45y; Long:3,886 (depression-free at baseline) | Living environmental factors (composite score: PM_2.5_, fuel type etc.) | Depressive symptoms (CESD-10 ≥10) | High vs low risk environment: cross-sectional depression OR=1.70, 95% CI:1.47-1.96, longitudinal HR=1.20, 95% CI:1.03-1.40; risk factors: solid fuel (HR=1.25), no piped water (HR=1.12) | Long-Term Exposure; High-Pollution Area; PM_2.5_; Indoor Solid Fuel |
| 56 | Yuan et al., 2025 | UK | Prospective cohort (UK Biobank) | 245,820; 37–73 y (mean56.6 y, SD=8.0) | Long-term ambient SO_2_ (annual mean <8μg/m^3^, low-concentration) | Incident mental disorders (depression/anxiety/BPD/SSD); Psychiatric symptoms | Per IQR SO_2_ increase (1.36μg/m^3^): depression HR=1.11, 95% CI:1.07-1.16, anxiety HR=1.10, 95% CI:1.06-1.14; SO_2_ 3–8 vs ≤3μg/m^3^: depression HR=1.25, anxiety HR=1.19, BPD HR=1.87; stronger in <60 years | Long-Term Exposure; Low-Pollution Area; Urban Environment; SO_2_ |
| 57 | Xiang et al., 2023 | China (28 Provinces) | Cross-sectional (CLASS 2018) | 11,418; ≥60 y (mean71.45 y) | Individual/regional factors (income, empty nest, air quality, GDP etc.) | Cumulative Health Deficit Index (CHDI) | Mental health indicators accounted for 75.73% of CHDI weight (depression/loneliness:33.37%; cognition:25.21%); CHDI showed Hu Line spatial heterogeneity; top factors: personal income (q=0.54), empty nest (q=0.52) | High-Pollution Area |
| 58 | Zhang & Wu, 2021 | China (25 Provinces) | Cross-sectional (CFPS 2016) | 4,997; ≥16 y (mean47.91 y, SD=16.75) | Community environment perception (6 dimensions, higher score=worse) | Depression (CESD score, 0–80) | Worse environment perception linked to higher depression scores (*β*=0.532, *p*<0.001); subjective social class mediated 5.45% of the association; stronger in males, rural residents, <60 years | High-Pollution Area; Urban Environment; Noise |
| 59 | Petkus et al., 2021 | USA (48 States) | Longitudinal cohort (WHIMS) | 6,118; ≥65 y (mean70.6 y, SD=3.8) | Long-term ambient PM_2.5_, NO_2_ (3-year annual average) | Global cognitive performance (3MS); Emotional distress | Per IQR increase: PM_2.5_ *β*=-0.144, 95% CI:-0.261 to -0.028, NO_2_ *ββ*=-0.157, 95% CI:-0.291 to -0.022 for 3MS; lower 3MS linked to more emotional distress; pollutants had indirect effects via 3MS | Long-Term Exposure; Low-Pollution Area; PM_2.5_; NO_2_ |
| 60 | Zhang, 2025 | China | Cross-sectional (chain mediation analysis) | 978; ≥60 y (60-69y:51.9%) | Environmental pollution perception (air/water/noise/sunlight) | Aging anxiety (physical/autonomy/economic) | Pollution linked to aging anxiety (direct effect *β*=0.092, 95% CI:0.010-0.173); mediated by healthcare visits (β=0.011) and sleep quality (*β*=0.016); significant chain mediation (pollution→visits→sleep→anxiety) | Long-Term Exposure; High-Pollution Area |
| 61 | Barnett et al., 2023 | Australia | Cross-sectional (mediation analysis) | 4,141; 34–97 y (mean61.1 y, SD=11.4) | Neighborhood environmental attributes (built/natural, PM_2.5_, NO_2_, SES) | HRQoL (SF-36 PCS/MCS) | Only neighborhood SES (β=0.202, 95% CI:0.092-0.312) and PM_2.5_ (β=-0.218, 95% CI:-0.393 to -0.044) had significant direct effects on SF-36 PCS; all attributes had indirect effects via physical activity/sedentary behaviour | Long-Term Exposure; Low-Pollution Area; Urban Environment; PM_2.5_; NO_2_; Green Space |
| 62 | Li et al., 2024 | China | Prospective cohort (CHARLS 2011–2018) | 9,452; ≥45 y (mean59.0 y, SD=9.8) | Cooking fuel type (consistent solid/clean, transition to clean) | Social participation (active vs isolated) | Clean fuel users (HR=1.31, 95% CI:1.19-1.44) and transitioners (HR=1.39, 95% CI:1.28-1.51) had higher active social participation; mediated by IADL (7.35%) and depression (1.82%) | Long-Term Exposure; High-Pollution Area; Indoor Solid Fuel |
| 63 | Zhang et al., 2022 | China (Rural) | Cross-sectional (ESR/ESP models) | 21,324; ≥16 y (mean45.85 y, SD=16.64) | Household non-traditional fuel use (gas/electricity) vs solid fuels | Depression (CESD-20 score; prevalence ≥16) | Non-traditional fuel users had lower CESD-20 scores (ATT=-3.659, *p*<0.01) and 8.2% lower depression probability (ATT=-0.082, *p*<0.01); mediated by reduced physical discomfort/chronic disease | Long-Term Exposure; High-Pollution Area; Rural Environment; Indoor Solid Fuel |
| 64 | Yu et al., 2021 | China | Longitudinal cohort (CHARLS 2011-2015) | 8,668; ≥45 y (64.6% ever used solid fuels) | Household solid cooking fuel use (consistent/inconsistent) | Sleep health (duration, feeling unrested) | Consistent solid fuel use: insufficient sleep OR=1.17, 95% CI:1.01-1.35, unrested ≥5d/week OR=1.32, 95% CI:1.12-1.55; stronger in ≥65 years; linked to sleep problems+depression comorbidity (OR=2.11, 95% CI:1.74-2.55) | Long-Term Exposure; High-Pollution Area; Indoor Solid Fuel |
| 65 | Gopinath et al., 2021 | Australia (Sydney, Blue Mountains) | Prospective cohort (BMES) | 1,085; ≥55 y | Visual/hearing/dual sensory impairment; hearing handicap | Successful aging (no disability/depression/cognition impairment/chronic disease) | Visual/hearing impairment vs none: OR=0.63, 95% CI:0.43-0.94 for successful aging; moderate hearing handicap OR=0.50; severe OR=0.39; no significant dual sensory impairment association | Long-Term Exposure; Low-Pollution Area |
| 66 | Lyons et al., 2024 | Ireland | Cross-sectional (retrospective cohort) | 3,407; ≥50 y (mean66.3 y, SD=8.4) | Long-term ambient PM_2.5_ (17-year annual average, 1km resolution) | Mental health (CESD-8, HADS-A, worry, stress, CASP-12) | Per 1μg/m^3^ PM_2.5_ increase: higher depressive symptoms (*β*=0.0312, 95% CI:0.0093-0.0531, *p*=0.0053) and anxiety (*β*=0.0380, 95% CI:0.0158-0.0602, *p*=0.0008); clinically significant anxiety OR=1.217, 95% CI:1.085-1.364; no association with worry, stress or QoL | Long-Term Exposure; Low-Pollution Area; PM_2.5_ |
| 67 | Chen et al., 2023 | China | Longitudinal panel (CHNS 2006,2009,2011,2015) | 7,514; ≥55 y (mean 65.8 y, SD=7.9) | Long-term ambient PM_2.5_ (annual average, city-level) | Mental health composite score (0–15; higher=better) | Per 10μg/m^3^ PM_2.5_ increase: 2.43-point mental health score decrease (*β*=-0.243, *p*<0.01, 2SLS); OLS showed weaker effect (*β*=-0.029); stronger in males, rural residents, low income/education; mediated by illness incidence (3.7%), severity (8.2%) and indoor reading (5.8%) | Long-Term Exposure; High-Pollution Area; PM_2.5_ |
| 68 | Liu et al., 2022 | China | Longitudinal panel (PSM-DID, fixed effects) | 5,526; ≥45 y | Cooking energy switch (solid vs clean: natural gas/electricity/biogas) | Depression (CESD score 0–100; binary >15); Cognitive ability (reasoning 0–11, memory 0–10) | Clean fuel switch linked to lower CESD (*β*=-1.52, *p*<0.01), lower depression odds (*β*=-0.019, *p*<0.05) and better reasoning (β=+0.14, *p*<0.05); no significant effect on memory | Long-Term Exposure; High-Pollution Area; Indoor Solid Fuel |
| 69 | Humphrey et al., 2024 | USA (New York City) | Time-stratified case-crossover | 837,523; median age=69 y (IQR=22) | Short-term ambient PM_2.5_, NO_2_, SO_2_, O^3^ (100m resolution, per 10-unit increase) | Acute cardiovascular events (ED visits/inpatient admissions: all CVD, IHD, HF, stroke, AMI) | NO_2_ (lag0): all CVD +1.51% (95% CI:1.22-1.80), IHD +1.89% (95% CI:1.34-2.45); PM_2.5_ (lag0): all CVD +1.09% (95% CI:0.71-1.47), HF +1.53% (95% CI:0.68-2.39); SO_2_: no effect; O^3^: inverse association | Short-Term Exposure; Low-Pollution Area; Urban Environment; PM_2.5_; NO_2_; O^3^; SO_2_ |
| 70 | Jang et al., 2021 | South Korea (Seoul/Incheon/Wonju/Pyeongchang/Gangwha) | Multicenter prospective cohort (EPINEF) | 3,775; ≥50 y (mean 68.8±6.7 y); neuroimaging subcohort=1,022 | Ambient PM_10_, PM_2.5_, NO_2_, Mn_10_, Mn_2.5_; urinary PAH metabolites (1-OHP, 2-naphthol) | Brain MRI markers (cortical thickness/volume); neuropsychological function (K-MoCA, RCFT, SVLT, SGDS-K) | PM_10_/NO_2_ linked to frontal/temporal cortical thinning and lower thalamus volume; 2-naphthol (males) associated with global/parietal/temporal/insular thinning; 1-OHP (females) with frontal/parietal thinning; 1-OHP linked to lower verbal learning/memory scores; no effect sizes reported | Long-Term Exposure; Low-Pollution Area; PM_2.5_; NO_2_; PM_10_; Mn |
| 71 | Andersen et al., 2024 | Denmark | Nationwide administrative cohort (Cox regression) | 934,792; ≥60 y (mean 71.8±8.3 y) | Long-term ambient PM_2.5_, NO_2_, BC (2010 LUR models, 100×100 m) | Incident dementia (first hospital contact/anti-dementia prescription, ICD-8/10) | Per IQR increase: PM_2.5_ (1.9μg/m^3^) HR=1.14, 95% CI:1.12-1.16, NO_2_ (10.2μg/m^3^) HR=1.25, 95% CI:1.22-1.28, BC (0.5×10^-5^/m) HR=1.23, 95% CI:1.20-1.26 for incident dementia; stronger in age >75, stroke history, low SES | Long-Term Exposure; Low-Pollution Area; PM_2.5_; PM_2.5_ Components; NO_2_ |
| 72 | Fu et al., 2025 | China (Sichuan, 9 Cities) | Time-stratified case-crossover | 7,282; mean 52.9±16.1 y (all ages) | Short-term ambient PM_2.5_, PM_10_, SO_2_, CO (daily, lags 0–7, moving averages 01–07) | Hospital admissions for anxiety disorders (ICD-10:F40/F41); economic burden | Per 10μg/m^3^: PM_2.5_ (lag5) OR=1.002, 95% CI:1.001-1.004, SO_2_ (lag5) OR=1.034, 95% CI:1.020-1.047; per 1mg/m^3^ CO (lag07) OR=1.614, 95% CI:1.247-2.089 for anxiety hospitalizations; PM-attributable economic cost=¥966,319; stronger in elderly and cold season | Short-Term Exposure; High-Pollution Area; PM_2.5_; PM_10_; SO_2_; CO |
| 73 | Zong et al., 2025 | China | Longitudinal cohort (double machine learning, instrumental variables) | 11,988; ≥45 y (45–60:61%, ≥60:39%) | HSR opening (binary), service frequency, network centrality | Global cognition (0–30), episodic memory, mental intactness | HSR opening linked to better global cognition (*β*=0.198, 95% CI:0.088-0.309) and mental intactness (*β*=0.111, 95% CI:0.053-0.170); no effect on episodic memory; mediated by PM_2.5_ (0.051), earnings (0.009) and depressive symptoms (0.038); stronger in women, urban, young-old, less-educated | Long-Term Exposure; High-Pollution Area; PM_2.5_ |
| 74 | Nguyen et al., 2021 | USA (California) | Two-stage time-series (quasi-Poisson) + meta-analysis | 1,997,992; all age groups | Short-term ambient O^3^ (8hr mean, per10ppb), PM_2.5_ (24hr mean, per10μg/m^3^, lags 0–7, cumulative 0–6/0–29) | ED visits for mental health (ICD-9:290–319) and subcategories (depression, self-harm, bipolar etc.) | O^3^ (lag0–6): all mental health ED visits +0.64% (95% CI:0.21-1.07), depression +1.87% (95% CI:0.62-3.15); PM_2.5_ (lag0): all mental health +0.42% (95% CI:0.14-0.70); stronger in females, Asians, Hispanics, children and warm season | Short-Term Exposure; Low-Pollution Area; PM_2.5_; O^3^ |
| 75 | Shen et al., 2021 | China | Longitudinal panel (2015/2018, city/year fixed effects) | 39,492 (physical health),17,250 (mental health); ≥45 y (mean 61.1 y) | Annual average AQI; PM_2.5_, PM_10_ (robustness check) | Physical health index, mental health index (cognition/memory/CESD), hospitalization costs | Per10-unit AQI increase: physical health index +0.72 (*β*=0.0723, *p*<0.01); mental health index -0.15 per1-unit AQI (*β*=-0.151, *p*<0.01); per1SD AQI increase: depression +1.032SD; hospitalization costs +19.06% per1-unit AQI; stronger in males | Long-Term Exposure; High-Pollution Area; Overall PM_2.5_; Composite Index (AQI/API) |
| 76 | Kumar et al., 2025 | Global (LMIC Focus, e.g., India) | Systematic review & meta-analysis + bibliometrics | 212; all ages (peak prevalence 55–65 y) | Solid cooking fuel emissions (PM_2.5_, PM_10_, CO, NO_2_, SO_2_) | Physical health (CVD, COPD, lung cancer, ARI); Mental health (cognitive decline, depression, anxiety, Alzheimer's) | PM_2.5_/PM_10_ strongest linked to CVD/COPD/lung cancer; children ARI: per10μg/m^3^ PM_2.5_ OR=1.06, 95% CI:1.05-1.07; all pollutants linked to cognitive decline/depression/anxiety; disease prevalence peaks at 55–65 years (I^2^=100%, *p*<0.001) | Long-Term Exposure; High-Pollution Area; PM_2.5_; Indoor Solid Fuel; NO_2_; PM_10_; SO_2_; CO |
| 77 | Sui et al., 2025 | Netherlands | Longitudinal relocation (PSM, DID) | 297; ≥18 y (mean 43.4 y) | Neighborhood environmental changes (≥10%: PM_2.5_, green/blue space, population density, deprivation, social fragmentation) | Mental health (MHI-5 score, 0–100; higher=worse) | Decreased PM_2.5_ (*β*=-3.869, 95% CI:-7.583 to -0.155), population density (*β*=-5.893) and deprivation (*β*=-4.756) linked to better mental health; increased social fragmentation (*β*=-3.520) also beneficial; green/blue space had no effect | Long-Term Exposure; PM_2.5_; Green Space |
| 78 | Zhou et al., 2023 | China (Wuhan) | Cross-sectional (WCDCS baseline) | 10,253; ≥18 y (mean 47.5 y) | Long-term ambient PM_10_, PM_2.5_, NO_2_, SO_2_, O^3^ (3-year average, nearest monitor) | Sleep disorder (≥3 days/week: insomnia, snoring; or hypnotic use) | Per IQR increase: PM_10_ aOR=1.147, 95% CI:1.062-1.240, PM_2.5_ aOR=1.089, 95% CI:1.003-1.182, SO_2_ aOR=1.158, 95% CI:1.065-1.260 for sleep disorder; NO_2_/O^3^ no effect; stronger in middle-aged/elderly and rural residents | Long-Term Exposure; High-Pollution Area; Urban Environment; PM_2.5_; NO_2_; O^3^; PM_10_; SO_2_ |
| 79 | Humphreys et al., 2022 | USA (Washington State, Methow Valley) | Exploratory qualitative (focus groups, key informant interviews) | 13 focus group (mean 46.6 y) +16 informants; ≥18 y | Extreme/persistent wildfire smoke events | Mental health/wellbeing (anxiety, depression, isolation); adaptation solutions | WFS linked to heightened anxiety, depression, isolation and physical health impacts; disproportionate effects on low-income, outdoor workers, elderly, preexisting conditions; proposed solutions: clean air spaces, air filters, stress reduction programs; no quantitative data | Low-Pollution Area; Rural Environment; Wildfire Smoke (PM_2.5_) |
| 80 | Li et al., 2021 | China (Nanjing) | Nested case-control (birth cohort) | 457; pregnant women (20–44 y, 22–24 weeks gestation); 313 high stress,144 controls | PM_2.5_ average daily dose (ADD, μg/kg·d) | High mental stress (Crown-Crisp phobic anxiety subscale ≥6) | Per1-unit ADD increase: OR=1.1376, 95% CI:1.0826-1.1953 for high mental stress (+13.76% risk); high stress group had higher ADD (10.55 vs7.87μg/kg·d, *p*<0.001) and perceived pollution effect (4.20 vs3.79, *p*<0.001); indoor attribution mediated the ADD-stress link | Short-Term Exposure; High-Pollution Area; PM_2.5_ |
